# A Hierarchical Multi-View Deep Learning Framework for Autism Classification Using Structural and Functional MRI

**DOI:** 10.3390/jimaging12030109

**Published:** 2026-03-04

**Authors:** Nayif Mohammed Hammash, Mohammed Chachan Younis

**Affiliations:** 1 Department of Computer Science, College of Computer Science and Mathematics, University of Mosul, Mosul 41002, Iraq; 2 Department of Computer Science, College of Computer Science and Mathematics, Tikrit University, Tikrit 34001, Iraq; 3 Department of Artificial Intelligent, College of Computer Science and Mathematics, University of Mosul, Mosul 41002, Iraq; mohammed.c.y@uomosul.edu.iq

**Keywords:** autism spectrum disorder, multi-view learning, hierarchical deep learning, spatiotemporal representation, cross-modality fusion

## Abstract

Autism classification is challenging due to the subtle, heterogeneous, and overlapping neural activation profiles that occur in individuals with autism. Novel deep learning approaches, such as Convolutional Neural Networks (CNNs) and their variants, as well as Transformers, have shown moderate performance in discriminating between autism and normal cohorts; yet, they often struggle to jointly capture the spatial–structural and temporal–functional variations present in autistic brains. To overcome these shortcomings, we propose a novel hierarchical deep learning framework that extracts the inherent spatial dependencies from the dual-modal MRI scans. For sMRI, we develop a 3D Hierarchical Convolutional Neural Network to capture both fine and coarse anatomical structures via multi-view projections along the axial, sagittal, and coronal planes. For the fMRI case, we introduced a bidirectional LSTM-based temporal encoder to examine regional brain dynamics and functional connectivity. The sequential embeddings and correlations are combined into a unified spatiotemporal representation of functional imaging, which is then classified using a multilayer perceptron to ensure continuity in diagnostic predictions across the examined modalities. Finally, a cross-modality fusion scheme was employed to integrate feature representations of both modalities. Extensive evaluations on the ABIDE I dataset (NYU repository) demonstrate that our proposed framework outperforms existing baselines, including Vision/Swin Transformers and various newly developed CNN variants. For the sMRI branch, we achieved 90.19 ± 0.12% accuracy (precision: 90.85 ± 0.16%, recall: 89.27 ± 0.19%, F1-score: 90.05 ± 0.14%, and focal loss: 0.3982). For the fMRI branch, we achieved an accuracy of 88.93 ± 0.15% (precision: 89.78 ± 0.18%, recall: 88.29 ± 0.20%, F1-score: 89.03 ± 0.17%, and focal loss of 0.4437). These outcomes affirm the superior generalization and robustness of the proposed framework for integrating structural and functional brain representations to achieve accurate autism classification.

## 1. Introduction

Autism Spectrum Disorder (ASD) is a complex neurodevelopmental disorder with persistent deficits in social interactions, communication, and restricted or repetitive behaviors and interests. From a neurobiological point of view, ASD is often linked to the atypical development of brain networks involved in social behavior, sensory processing, and executive control. Neuroimaging studies have repeatedly shown structural and functional differences in regions such as the medial prefrontal cortex, posterior cingulate cortex, temporal lobes, and cerebellum, along with altered long-range connectivity between frontal and posterior areas [1]. Early identification of ASD is crucial, as timely intervention for individuals with autism can improve progressive outcomes and quality of life [2].

In recent years, Magnetic Resonance Imaging (MRI) has become a vital modality for research into neurological and psychiatric disorders, providing a non-invasive window into brain structure and function. Among the various types of MRI, structural MRI (sMRI) and functional MRI (fMRI) are the most widely used and complementary methods [3]. sMRI provides high-resolution anatomical detail, including cortical thickness, gray–white matter composition, and volumetric discontinuities, allowing researchers to identify low-level morphological abnormalities. Meanwhile, fMRI captures the blood-oxygen-level-dependent (BOLD) signal, enabling researchers to analyze functional connectivity and dynamic interactions between brain regions [4]. While sMRI highlights static structural aspects, fMRI reveals neural activity over time and provides a more easily understandable view of both. In ASD diagnosis, various researchers report atypical brain morphology and disrupted functional connectivity across studies. Therefore, performing a joint analysis of sMRI and fMRI provides a more comprehensive understanding than either modality alone. This complementary nature has drawn significant attention to deep learning fusion frameworks [5], which leverage detailed anatomical information from sMRI and combine it with dynamic functional mapping of brain states from fMRI to enhance the accuracy of the outcome modality and clinical interpretability.

Despite their clinical relevance, MRI-based approaches face challenges due to the large number of slices acquired per subject, particularly in high-resolution structural and dynamic functional MRI. This increased volume of data substantially raises clinician workload and, in turn, the risk of diagnostic errors [6]. The second challenge is inter-protocol variability, scanner variation, and the requirement for precision in slice-wise or voxel-wise interpretation [7]. This step introduces substantial delays and may lead to errors in manual diagnosis. The third challenge is that many ASD-related abnormalities exhibit subtle, diffuse, and heterogeneous patterns of neurological dysfunction variability [8]. For example, while sMRI detects fine-grained deviations in animal structure, such as cortical thickness, gray matter density, and volumetric changes, fMRI detects temporal changes and problems in the functional connectivity within brain networks. Because these abnormalities exhibit substantial variability across individuals, consistent early diagnosis becomes difficult with expert assessment alone. As a result, there has been a significant increase in interest in using machine learning (ML) and deep learning (DL) frameworks that leverage the complementary aspects of sMRI and fMRI data, combining them for the automated detection and description of ASD [9].

Traditionally, ML-based approaches have relied on handcrafted features derived from sMRI or fMRI, which are then subsequently classified using traditional classifiers, such as support vector machines (SVMs), random forests, or logistic regression [10,11,12]. There were several shortcomings with this method, as the limited, manually engineered features contributed to its generalizability. More recently, DL-based models have gained popularity for ASD diagnosis, utilizing methods such as convolutional neural networks (CNNs) [13,14,15], autoencoders [16], and transformer-based attention mechanisms [17,18] to facilitate end-to-end learning. These approaches effectively extract hierarchical features directly from the raw neuroimaging data, thereby bypassing the need for manual feature engineering. However, a key limitation across many existing studies is their reliance on small, single-modality datasets, which can lead to overfitting and reduce the robustness of results across heterogeneous clinical populations [19].

To address these issues, dual-modality learning frameworks incorporating both sMRI and fMRI have recently been investigated. Such models have the potential to incorporate structural and functional information and will be much better equipped to capture the multidimensional neural correlates of ASD. However, dual-modality approaches also pose considerable challenges, including feature alignment, high-dimensional feature fusion, and generalization, due to the potential to be trained with fewer samples. These shortcomings underscore the need for advanced, effective DL architectures that can effectively leverage sMRI and fMRI in a unified manner to address dataset constraints and improve clinical utility.

In this work, we present a hierarchical multi-view deep learning framework that uses sMRI and fMRI datasets for ASD classification. To leverage the complementary information in the MRI data, each subject’s scans were pre-processed to include three orthogonal anatomical views: axial, coronal, and sagittal. The three multi-view slices for each subject were matched to a common standardized spatial resolution, resized to a common volumetric aspect ratio, and assembled into 3D tensors to form a single input for our model. The backbone architecture of our model is a newly introduced hierarchical 3D convolution that learns progressively from shallow to high-level volumetric features. The framework comprises different stages, including convolutional layers, normalization, pooling, and adaptive weighting, to capture both the gradual loss of underlying morphological features, such as the local convexity of certain shapes, and the diffuse disruptions in connectivity of the overall brain. Moreover, we integrated Focal Loss with adaptive weighting to address imbalances in the distributions of sMRI and fMRI slices, thereby providing a more robust training approach. Finally, we incorporated the Mixup augmentation strategy into our framework to enhance model generalization by initializing the model with a weighted combination of samples.

Our contribution can be summarized as follows.

1. We develop a unified multi-view preprocessing pipeline for sMRI and fMRI that ensures consistent spatial and temporal representation across modalities.
2. We introduce a hierarchical feature extraction framework that captures shallow intermediate and deep representations with Grad-CAM-based interpretability.
3. We propose a voxel-level temporal modeling strategy that uses an LSTM encoder to learn dynamic patterns of brain activity.
4. We design an attention-driven cross-modality fusion mechanism that integrates spatial and temporal cues for robust joint feature representation.
5. We construct a multilayer perceptron-based decision network that provides reliable classification of autism versus control with complete end-to-end interpretability.

## 2. Related Works

Several research studies have investigated various potential methods and neuroimaging approaches for diagnosing ASD (Table 1). These methods aim to integrate preprocessing, segmentation, and classification pipelines to enhance diagnostic accuracy while accounting for both the complexity of brain changes associated with ASD and the associated neuroimaging. For example, Subah et al. (2021) [20] tackled the limitations of small datasets in fMRI studies and the poor generalizability of ASD detection. They proposed a deep neural network incorporating functional connectivity features from various brain atlases, including CC200, AAL, BASC, and Power. The model demonstrated a mean accuracy of 88%, with 90% sensitivity, an F1-score of 87%, and an AUC of 96%, exceeding the accuracy of previous methods. One limitation is reliance on a selected atlas. In the present study, the BASC was the most superior atlas, but reliance on any specific atlas may impact generalizability to other datasets. Mostafa and colleagues (2023) [21] tackled the early diagnostic issue of ASD, which currently lacks reliable biomarkers or standardized medical assessments. They developed a deep learning framework that utilized fMRI and sMRI images, eliminating the need for complex data pre-processing using the ABIDE dataset. This architecture achieved accuracies of 80–84%, which were higher than those of the existing processing.

Jain et al. (2023) [22] proposed a DM-ResNet framework for classifying individuals with ASD from their MRI scans that combined hybrid Fuzzy C Means (FCM) with Gaussian Mixture Model (GMM) segmentation, features from VGG-16, and Dwarf Mongoose optimization. The authors reported an accuracy of 99.83% which represents a dramatic improvement in diagnosis from MRI scans. The pipeline itself combines preprocessing, segmentation, and optimized deep learning classification. However, the results so far, which are not aligned with baseline diagnostic capabilities, raise questions about the method’s robustness and generalizability when applied to different clinical datasets. Park & Cho (2023) [23] proposed a two-step deep learning model to diagnose ASD using functional connectivity signals between the superior temporal sulcus and the visual cortex. The model uses a residual attention network to capture spatiotemporal features, in conjunction with a graph convolutional network for ASD classification based on identified connectivity graphs. When using ABIDE fMRI data for testing, the authors conducted 10-fold cross-validation, and their model’s performance outperformed state-of-the-art approaches by 11.37%. Although the proposed method demonstrates accuracy, relying on specific connectivity hypotheses may limit generalizability across broader clinical samples.

Zhang et al. (2023) [24] proposed method is evaluated on the worldwide fMRI dataset, known as ABIDE (Autism Brain Imaging Data Exchange). The fMRI functional connectivity features selected using our method can achieve an average accuracy of 64.53% on intra-site datasets and an accuracy of 70.9% on the whole ABIDE dataset. Khudri et al. (2024) [25] developed a pipeline that included preprocessing fMRI data, extracting cortical surfaces, parcellating the brain into regions, estimating a correlation matrix, selecting features via recursive feature elimination, and classifying using a linear support vector machine. They tested the model on the ABIDE II dataset and achieved 97% accuracy, 90% sensitivity, and 99% specificity. One limitation of their approach is the requirement to use parcellation schemes and radiomic features, which limits the degree to which the method can be adapted to different cohorts.

Gulati et al. (2024) [26] addressed the problem of early detection of ASD using high-dimensional resting-state fMRI data. They proposed a neural network architecture based on a CNN that combines convolution, pooling, normalization, dropout, and dense layers, and trained it on ABIDE I samples. The model achieved impressive performance across multiple measures, with 99.39% accuracy, 98.80% recall, 99.85% precision, and an F1 score of 99.32%. A limitation of the study is its relatively homogeneous sample, which raises questions about its generalizability to a broader population. Wang et al. (2025) [27] investigated glymphatic function and white matter integrity in children with ASD using multi-parametric MRI and a machine-learning approach to support diagnostic evaluation. They extracted various indices, including aDTI-ALPS, fractional anisotropy, cerebrospinal fluid volume, and perivascular space volume, to feed into an AutoGluon-based model. This approach achieved an AUC of 0.84 with significant group differences and correlations to ASD symptom severity and developmental history. The study is limited by its design, specifically its retrospective nature and the small validation cohort across two centers. All studies above are summarized in Table 1 below.

## 3. Proposed Methodology

**Figure 1:**
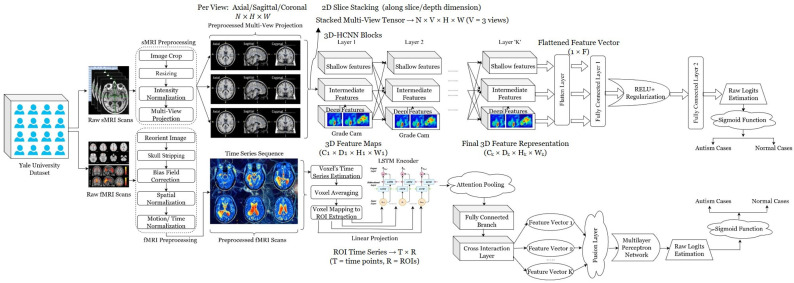
Comprehensive Hierarchical Multi-View Deep Learning Framework for Dual-Modal MRI Classification.

In this research, we propose a deep learning framework that jointly uses structural MRI (sMRI) and functional MRI (fMRI) to identify autism. sMRI scans undergo modality-specific preprocessing, including image cropping, resizing, intensity normalization, and multi-view projection (axial/sagittal/coronal). In contrast, fMRI scans undergo reorientation, skull stripping, bias field correction, spatial normalization, motion/time normalization, and voxel-wise time-series extraction. Spatial features are learned from multi-view sMRI using convolutional feature extractors, and temporal dynamics are extracted from fMRI using recurrent encoders with attention pooling. Modality-specific representations are fused via cross-interactions and relayed to fully connected layers, with some regularization, to generate a sigmoid output that distinguishes autism from typical cases. The working pipeline of the proposed architecture is shown in Figure 1.

### 3.1. Preprocessing

The preprocessing phases constitute a structured sequence of steps designed to produce high-quality, normalized input, ensuring comparable processing across both sMRI and fMRI modalities. Structural MRI preprocessing, including brain extraction, intensity normalization, and spatial registration to the MNI152 template, was performed using Python-based libraries with NiBabel 5.2 for MRI data handling. Functional MRI preprocessing, including motion correction, framewise displacement estimation, temporal filtering, and ROI time-series extraction, was carried out using established neuroimaging toolkits. The details of the applied preprocessing steps are given below:

#### 3.1.1. Brain Extraction

Raw image intensity at voxel x is denoted by I(x). A binary brain mask B(x) is computed to remove non-brain tissues using Equation (1).

(1) $$I_{Brain}\left(x\right)=I\left(x\right).B(x)$$

#### 3.1.2. Intensity Normalization

To ensure uniform contrast across subjects, voxel intensities are normalized using z-score normalization (Equation (2)).

(2) $$I_{Norm}\left(x\right)=\frac{I_{Brain}\left(x\right)-\mu }{\sigma }$$

where μ and σ are the mean and standard deviation of intensities for each scan.

#### 3.1.3. Spatial Registration

Each image is aligned to the standard MNI152 template by estimating a transformation matrix T that maximizes mutual information (MI) between the normalized subject image ($I_{Norm}$) and template ($I_{template}$) using Equation (3).

(3) $$T^{*}=\underset{T}{\mathrm{argmax MI}}\left(I_{Norm}⊙T,\;I_{template}\right)$$

#### 3.1.4. fMRI Motion and Temporal Correction

For each moment in time *t*, rigid-body motion parameters ($∆x_{t},\;∆y_{t},\;∆z_{t},\;∆\alpha_{t},\;∆\beta_{t},\;∆\gamma_{t}$) are estimated, where $∆x_{t},\;∆y_{t},$ and $∆z_{t}$ denote translational displacements along the x, y, and z-axes, and $∆\alpha_{t},\;∆\beta_{t},$ and $∆\gamma_{t}$ represent rotational displacements about the corresponding axes between consecutive fMRI volumes. With these parameters, framewise displacement (FD) is computed using Equation (4).

(4) $${FD}_{t}=\left|∆x_{t}\right|+\left|∆y_{t}\right|+\left|∆Z_{t}\right|+r(∆\alpha_{t}+∆\beta_{t}+∆\gamma_{t})$$

where r is the brain radius (≈50 mm). Volumes exceeding the threshold FD are discarded. Additionally, Temporal band-pass filtering is applied to the fMRI time series to remove low-frequency drift and high-frequency physiological noise. Let S(t) denote the original fMRI signal at time index t, and B(t) represent the filtered signal. The filtering retains frequency components within the range defined by the lower and upper cutoff frequencies $f_{l}$ and $f_{h}$, respectively. In this study, $f_{l}$ = 0.01 Hz and $f_{h}$ = 0.1 Hz are used to preserve neurophysiologically relevant BOLD fluctuations while suppressing noise outside this band. The implementation is defined in Equation (5).

(5) $$S_{filterd}(t)=\sum_{f=f_{l}}^{f_{h}}\hat{S}(f)e^{2\pi ft}$$

#### 3.1.5. Region of Interest Time Series and Connectivity (fMRI)

Region of Interest (ROI) refers to anatomically or functionally relevant brain regions extracted from the input images to focus the analysis on informative areas while suppressing background noise. In slice-based processing, ROI representations aggregate region-specific features while suppressing non-brain and background regions, enabling more robust, region-aware feature learning.

For a given brain parcellation into N ROIs, the mean fMRI time series for the region r is computed by averaging the voxel-wise BOLD signals within that region. Let $V_{r}$ denote the set of voxels belonging to ROI r, and $S_{v}\left(t\right)\;$ represent the BOLD signal intensity at voxel v ∈ $V_{r}$ at time index t. The regional time series $X_{r}(t)$ is obtained by computing the mean signal across all voxels in $V_{r}$, thereby yielding a representative temporal activity profile for each ROI while reducing voxel-level noise using Equation (6).

(6) $$s_{v}\left(t\right)=\frac{1}{\left|v_{r}(t)\right|}\sum_{v\in V_{r}}S_{v}(t)$$

Functional connectivity between regions i and j is quantified via Pearson correlation using Equation (7)

(7) $$r_{ij}=\frac{\sum_{t}\left(s_{i}\left(t\right)-{\bar{s}}_{i})(s_{i}\left(t\right)-{\bar{s}}_{j}\right)}{\sqrt{\sum_{t}{\left(s_{i}\left(t\right)-{\bar{s}}_{i}\right)}^{2}\sum_{t}{\left(s_{i}\left(t\right)-{\bar{s}}_{j}\right)}^{2}\;}}$$

and variance-stabilized using Fisher’s transform given in Equation (8).

(8) $$z_{ij}=tanh^{-1}\;(r_{ij})$$

Slices were saved in PNG format to enable efficient storage, consistent intensity scaling, and direct compatibility with 2D convolutional backbones, while preserving relative anatomical contrast after normalization.

### 3.2. Feature Extraction and Classification

Following the preprocessing of sMRI and fMRI data, the next crucial step is to extract meaningful features and learn robust representations from both modalities. In the experiment, the number of slices was varied from 10 to 50 to study the trade-off between anatomical coverage and computational efficiency. Lower slice counts capture only limited local context, while higher slice counts provide broader anatomical representation at the cost of increased redundancy and memory usage. We employ a hybrid feature-extraction scheme because sMRI and fMRI contain complementary structural and functional information.

#### 3.2.1. sMRI Feature Extraction

For the structural MRI scans, we first produce multi-view projections (axial, sagittal, and coronal) to maintain anatomical details across the orientations. These projections undergo a deep learning hierarchical pipeline. The proposed 3D Hierarchical Convolutional Neural Network (3D-HCNN) extracts the inherent spatial dependencies in these projections while retaining hierarchical patterns across multiple scales.

Given an input sMRI voxel projection defined as $X\in R^{H\times W\times D\times C}$, where H, W, D denote the spatial dimensions and C = 1 corresponds to the modality channel, the network defines a mapping rule: $f_{\theta }:X\mapsto Z$ where $Z\in R^{d}$ refers to the discriminative latent embedding. Feature learning starts with a 3D convolutional layer, which works on local voxel neighborhoods, creating a transformed feature map, calculated as:

(9) $$F^{l}=\sigma (B(W^{l}*F^{l-1}+b^{l})),$$

where $W^{l}\in R^{k\times k\times k\times C_{in}\times C_{out}}$ is a 3D kernel with a size of k×k×k, ∗ denotes convolution, $b^{l}$ is the bias term, B denotes normalization, and σ(.) refers to a nonlinear activation function. Kernel sizes k = {3, 5} are alternated across layers to achieve a trade-off between fine and coarse anatomical encoding, and the number of output channels is subsequently increased in a hierarchical order of {32, 64, 128, 256}. To accommodate variable receptive fields, dilated convolutions are used, with the convolution kernel operating at a dilation rate d. The operation dilated at voxel v can be expressed by Equation (10).

(10) $$\left(F^{\left(l\right)}{*}_{d}w^{\left(l\right)}\right)\left(v\right)=\sum_{u}F^{\left(l-1\right)}\left(v+d.u\right)W^{\left(l\right)}\left(u\right)$$

allowing the model to capture both granular cortical features and large-scale anatomical variability without exponentially increasing the number of parameters.

Each block is further stabilized with spectral normalization, which constrains the Lipschitz constant of convolution operators: if the convolution kernel is reshaped as a matrix W, spectral normalization rescales the matrix W based on the maximum singular value ($\sigma_{max}(W)$) using Equation (11).

(11) $$\hat{W}=\frac{W}{\sigma_{max}(W)}$$

This ensures stable gradient propagation and prevents exploding activations in deep hierarchical layers. To adaptively modulate features, statistical normalization is applied. For a feature map $F^{l}$, with the total number of voxels (N=H×W×D), mean and variance are calculated as follows:

(12) $$\mu^{l}=\frac{1}{N}\sum_{i=1}^{N}F_{i}^{l},\;\sigma^{2l}=\frac{1}{N}\sum_{i=1}^{N}{\left(F_{i}\left(l\right)-\mu^{l}\right)}^{2}$$

and features are normalized and rescaled through the following Equation.

(13) $$\hat{F^{l}}=\gamma^{\left(l\right)}*\frac{\left(F_{i}\left(l\right)-\mu^{l}\right)}{\sqrt{\sigma^{2l}+\epsilon }}+\beta^{l}$$

where $\gamma^{\left(l\right)}$ and $\beta^{l}$ are affine learnable parameters. To enable gradient flow and provide more capacity for modeling deeper architectures, residual connections are introduced between hierarchical blocks. The residual fusion in layer *l* can be written as

(14) $$R^{\left(l\right)}=F^{\left(l\right)}+\emptyset (F^{\left(l-1\right)})$$

with *ϕ*(⋅) being a 1 × 1 × 1 convolution for dimensional alignment. In addition to convolutional feature encoding, a channel–spatial attention mechanism is incorporated to enhance alertness to discriminative anatomical regions. For a feature tensor $F\in R^{H\times W\times D\times C}$, channel attention weights are computed based on a softmax-normalized projection given in Equation.

(15) $$\alpha_{c}=\frac{\exp\left(w_{c}^{T}g(F)\right)}{\sum_{j=1}^{C}w_{j}^{T}g(F)}$$

where *g*(*F*) is a global pooling function and $w_{c}$ are learnable parameters. The reweighted representation becomes

(16) $$F^{\prime }=\sum_{c=1}^{C}\alpha_{c}.F_{c}$$

while spatial attention is enforced by a 3D sigmoid mask $M\in {\left(0,1\right)}^{\left(H\times W\times D\right)}$ such that

(17) $$F^{\prime \prime }=F^{\prime }⊙M$$

**Figure 2:**
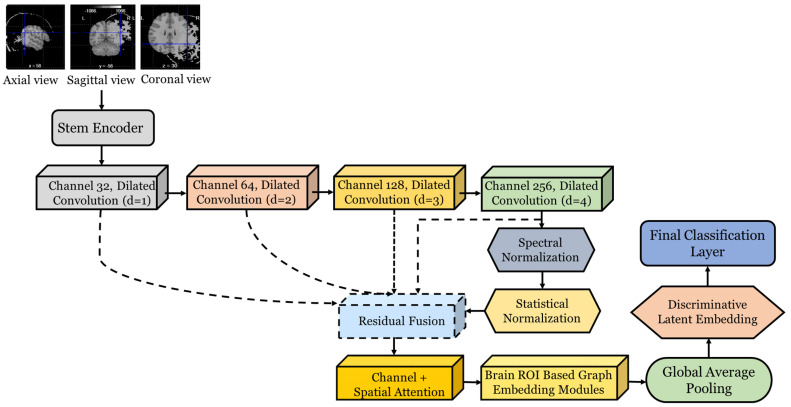
Detailed Architecture of the sMRI Feature Extractor.

To ensure anatomical consistency, the features are regularized using a graph-based embedding module. Let us consider the brain parcellated into *n* regions-of-interest (ROIs), with the ROIs represented as nodes V = $\left\{v_{1},v_{2},\ldots v_{n}\right\}$ in a graph G = (V,E). The adjacency matrix $A\in R^{n\times n}$ encodes structural proximity or functional correlations among ROIs. The feature propagation at layer l is expressed as:

(18) $$H^{\left(l+1\right)}=\sigma (AH^{\left(l\right)}W^{\left(l\right)})$$

where *H*(*l*) is the ROI-level embedding and *W*(*l*) is a learnable weight matrix. The final feature vector is compacted via global average pooling:

(19) $$z=\frac{1}{HWD}\sum_{i=1}^{H}\sum_{j=1}^{W}\sum_{k=1}^{D}F_{\left(i,j,k\right)}^{\left(L\right)}$$

and constrained with an entropy minimization term in order to encourage compactness of features. The working pipeline of the sMRI-based feature extraction framework is illustrated in Figure 2.

#### 3.2.2. fMRI Feature Extraction

Following preprocessing, the fMRI data are summarized at the ROI level. For a given ROI *r*, let the mean BOLD activity at time step *t* be calculated as follows:

(20) $$s_{t,r}=\frac{1}{V_{r}}\sum_{v\in V_{r}}X_{pre}(v,t)$$

where $V_{r}$ is the set of voxels within region *r*. Performing this aggregation step converts a noisy voxel-level signal into a more interpretable regional-level activity signal. By collecting these ROI signals across all regions, the temporal evolution of the brain is represented as

(21) $$S=[s_{t,1},s_{t,2},\ldots ,s_{t,R}]\;\in R^{T\times R}$$

where the rows indicate one time step, and the columns represent one ROI. The matrix is the basis for examining how brain regions interact over time. To reduce redundancy and emphasize latent activity patterns, the matrix is projected into a lower–dimensional space,

(22) $$H_{t}=S_{t}W_{p}+b_{p}\;\;\;H\in R^{T\times d_{h}}$$

where $W_{p},b_{p}$ are learnable weights and biases, and $d_{h}$ is the embedding dimension. This representation captures compact temporal descriptors of brain activity. The temporal sequence *H* is then modeled by a bidirectional LSTM. The forward and backward passes are as follows:

(23) $${\vec{h}}_{t}={LSTM}_{f}\left(H_{t},{\vec{h}}_{t-1}\right),\;\;{\overset{\leftarrow }{h}}_{t}={LSTM}_{b}\left(H_{t},{\overset{\leftarrow }{h}}_{t+1}\right)$$

with their concatenation producing

(24) $$h_{t}=[{\vec{h}}_{t};{\overset{\leftarrow }{h}}_{t}]$$

The bidirectionality ensures that both past and future temporal contexts are captured. From a clinical viewpoint, this is critical, as atypical connectivity in autism manifests as disrupted temporal coordination, which simple models of either forward or reverse cannot capture. Given that every time slip contributes equally to diagnostic information, attention is focused on the relevant parts. Each time step’s attention weight is calculated as

(25) $$\alpha_{t}=\frac{exp(w^{T}h_{t})}{\sum_{\tau =1}^{T}exp(w^{T}h_{t})}$$

and the sequence–level embedding is obtained by

(26) $$Z_{seq}=\sum_{t=1}^{T}\alpha_{t}h_{t}$$

This implies that the model automatically places more focus on important patterns, in this case, abnormal synchronization events involving the social brain network, while disregarding irrelevant fluctuations. The embedding is also processed using a nonlinear transformation,

(27) $$f_{seq}=\sigma (W_{f}z_{seq}+b_{f})$$

where σ(⋅) is a ReLU activation function. In parallel, functional connectivity (FC) features, including correlations between ROIs, can be extracted to characterize inter–regional relationships. These are projected into the same latent space using Equation (28).

(28) $$f_{fc}^{\prime }=W_{fc}f_{fc}+b_{fc}$$

The interaction between sequential dynamics and connectivity is then modeled as

(29) $$f_{cross}=f_{seq}⊙f_{fc}^{\prime }$$

where the element-wise multiplication describes how the regional time-varying activity aligns with stable connectivity patterns. Finally, the sequence embedding, connectivity features, and their interaction are combined into a joint representation to fuse temporal, spatial, and relational information using Equation (30).

(30) $$f_{concat}=[f_{seq},f_{fc},f_{cross}]$$

**Figure 3:**
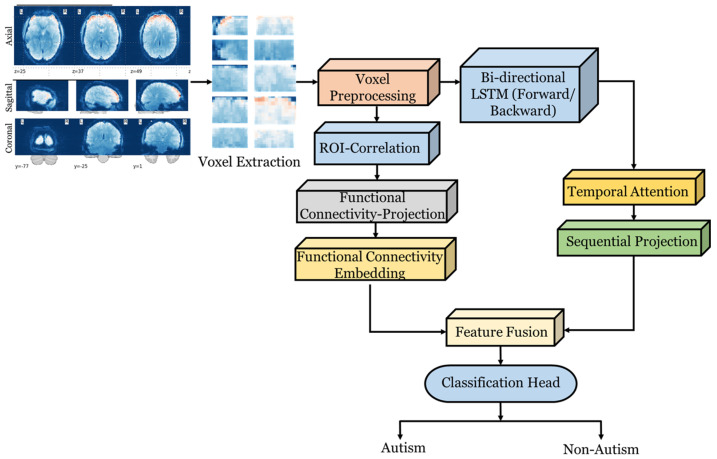
Architecture of the fMRI Feature Extractor with Spatiotemporal Modeling.

This hybridized feature vector is passed into the classification phase to determine the probability of either an autism or a non-autism case. The working pipeline of the fMRI-based feature extraction framework is illustrated in Figure 3.

#### 3.2.3. Classification

The feature vector produced by each modality-specific backbone (fMRI branch/the sMRI branch) is processed by an identical multilayer perceptron (MLP) classifier across both phases of the model [28] to ensure architectural uniformity and comparability between modalities. Let the extracted feature vector be denoted as $f\in R^{d}$, where *d* is the modality-specific feature dimension. A layer normalization (LN) is applied to this feature vector to ensure a consistent feature scale across subjects prior to projection, using Equation (31).

(31) u=LayerNorm(f)

The normalized representation is fed into the first dense layer, which has 256 neurons, where the transformation is defined as

(32) $$h^{\left(1\right)}=\phi (uW^{\left(1\right)}+b^{\left(1\right)}),\;W^{\left(1\right)}\in R^{d\times 256}b^{\left(1\right)}\in R^{256}$$

Here, *ϕ*(·) represents the Gaussian Error Linear Unit (GELU) activation function. We selected GELU because its smooth, non-linear properties approximate stochastic neuron firing more closely than ReLU. The model also applies a dropout layer with probability *p* = 0.5 to $h^{\left(1\right)}$, yielding a regularized hidden representation ${\tilde{h}}^{\left(1\right)}$.

The second-to-last stage of the MLP is a linear layer that maps this 256-dimensional representation to two output logits for the diagnostic classes:

(33) $$o=(h^{\left(1\right)}W^{\left(2\right)}+b^{\left(2\right)}),\;W^{\left(2\right)}\in R^{256\times 2},\;b^{\left(2\right)}\in R^{2}$$

The logits are used as inputs to the loss function (cross-entropy or focal loss), with no activation applied in the network itself. In the evaluation task, logits are passed through a softmax function externally to convert them into class probabilities.

(34) $$\hat{y}=softmax(o)$$

By using the same MLP classifier with both fMRI and sMRI features, the framework enables the evaluation of discriminative power in each modality under the same classification conditions.

## 4. Results and Discussion

This section presents the results and discussion of the experiment using the proposed multimodal deep learning framework for autism classification. The evaluation focuses on the accuracy of diagnosis, the model’s robustness, and its explainability when applied to structural and functional MRI modalities. The details of the experimental setup, dataset preparation, and used performance metrics are covered in the subsequent subsections.

### 4.1. Dataset Description

Magnetic Resonance Imaging (MRI) is a non-invasive neuroimaging modality that provides crucial information on cognitive biomarkers to diagnose neurological and neurodevelopmental conditions, such as schizophrenia, autism spectrum disorder (ASD), and Alzheimer’s disease [29]. Depending on the method of acquisition, MRI is classified as either a functional or a structural modality. Structural MRI (sMRI) provides not only angiometric (volume-type) measurements but also morphometric measurements of neuroanatomical abnormalities in the axial, coronal, and sagittal planes [30]. sMRI is more commonly used in clinical studies because it is effective at detecting subtle structural changes and producing high-contrast, high-resolution images [31]. Functional magnetic resonance imaging (fMRI) measures brain function by detecting changes in blood flow in response to neural activity in distinct brain areas. Functional MRI data represent the brain as a complex of voxels, which represent units of observation, and each voxel indexes changes in neural activation through the varying intensities of the received temporal signals [25,28].

In this study, we utilize the New York University (NYU) neuroimaging dataset [28], which was collected from the publicly available Autism Brain Imaging Data Exchange (ABIDE I) repository. The use of the NYU site alone reduces inter-site confounds but limits exposure to scanner and protocol variability, which may affect generalization to data acquired at other sites. The statistical overview of the NYU dataset is given in Table 2.

**Figure 4:**
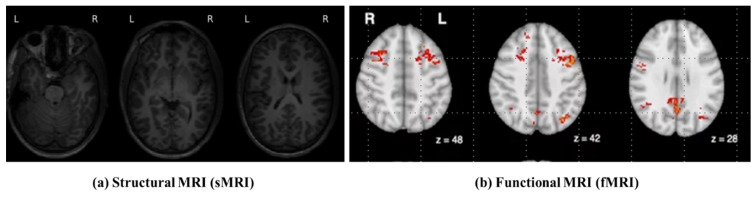
Visual Representation of Dual-Modal Neuroimaging Data from the NYU Dataset. (**a**) Samples of Structural MRI (sMRI) scans, (**b**) Samples of functional MRI (fMRI) scans collected from the NYU dataset. The red color region refers the activated BOLD regions in fMRI imaging.

The dataset was preprocessed using standard ABIDE workflows, including motion correction, skull stripping, slice-timing correction, spatial normalization to the MNI152 template, and temporal filtering to remove low-frequency drift. We restricted subjects to only those with complete phenotypic and imaging information to ensure consistency and reliability in our evaluations. A few samples of sMRI and fMRI scans are illustrated in Figure 4.

### 4.2. Slice Extraction from sMRI Modalities

Recently, 3D Convolutional Neural Networks (3D-CNNs) have emerged as an effective approach for analyzing volumetric structural MRI (sMRI) data. However, appropriate preprocessing is required to ensure spatial consistency and uniform input dimensions. Each sMRI scan is stored in Neuroimaging Informatics Technology Initiative (NIfTI) format (.nii) and represents the brain as a volumetric matrix composed of multiple two-dimensional (2D) slices organized along three anatomical planes: axial, coronal, and sagittal, as illustrated in Figure 4. These complementary planes collectively capture morphological patterns relevant to Autism Spectrum Disorder (ASD) [30].

The raw sMRI volumes are loaded and processed using Nibabel, a Python-based neuroimaging library. A typical sMRI scan contains approximately 128–256 slices per anatomical plane, which introduces substantial redundancy and computational overhead. Although ASD is a whole-brain disorder, ASD-related alterations are predominantly observed in central cortical and subcortical regions, which are well represented in the middle slices of the MRI volume. In contrast, peripheral slices often contain limited discriminative information while increasing computational complexity. Accordingly, central slices are adopted as a computationally efficient approximation, enabling effective feature learning without full volumetric modeling.

**Figure 5:**
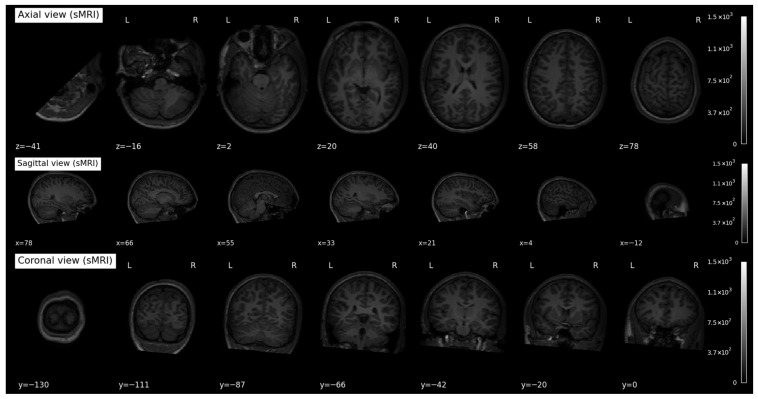
Visualization of the Multi-View Projection and Data Augmentation Strategy for sMRI.

To generate standardized input volumes for 3D-CNN data architecture, three slice-extraction methods were developed, and the resulting data are summarized in Table 3. In the first procedure, a single mid-slice was selected along each anatomical plane—axial, coronal, and sagittal—corresponding to the central coordinate of (x, y, z/2), or (x, y/2, z), and (x/2, y, z) mid-slice coordinates correspondingly. These mid-slices were resized to 224 × 224 pixels and saved as PNGs for visualization and quality checks. For the second and third procedures, 10 and 50 successive mid-slices were extracted from each plane, respectively, to enhance the local continuity of anatomical structure and inter-slice correlations needed for 3D-CNN learning. The slices were concatenated and provided to the volumetric tensors, which were then reshaped to a constant shape for training the neural network. To improve robustness and reduce overfitting, data augmentation was applied during training. Augmentation operations included random in-plane rotations within ±10°, horizontal and vertical flips with a probability of 0.5, and slight intensity perturbations using scaling factors in the range [0.9, 1.1]. For fMRI-derived slices, augmentation was applied consistently across slices from the same volume to preserve temporal coherence. No augmentation was applied during validation or testing. The train–test partitioning was conducted at the subject level prior to any augmentation, ensuring that no augmented or derived samples from a given subject appeared in the test set. Figure 5 provides examples of the augmented slices from each anatomical plane. This preprocessing pipeline was designed to handle spatially coherent, anatomically rich input data, enabling the 3D-CNN to learn features across anatomically distinct brain regions.

### 4.3. Slice Extraction from fMRI Modalities

Before using fMRI data for model fitting, a preprocessing pipeline was applied to generate 3D brain volumes that reflect functional brain activity associated with ASD. Each subject’s fMRI scan was initially treated as a 4D volume, comprising three spatial dimensions and one temporal dimension. All original temporal frames were retained and processed sequentially to preserve the temporal dynamics of brain activity.

**Figure 6:**
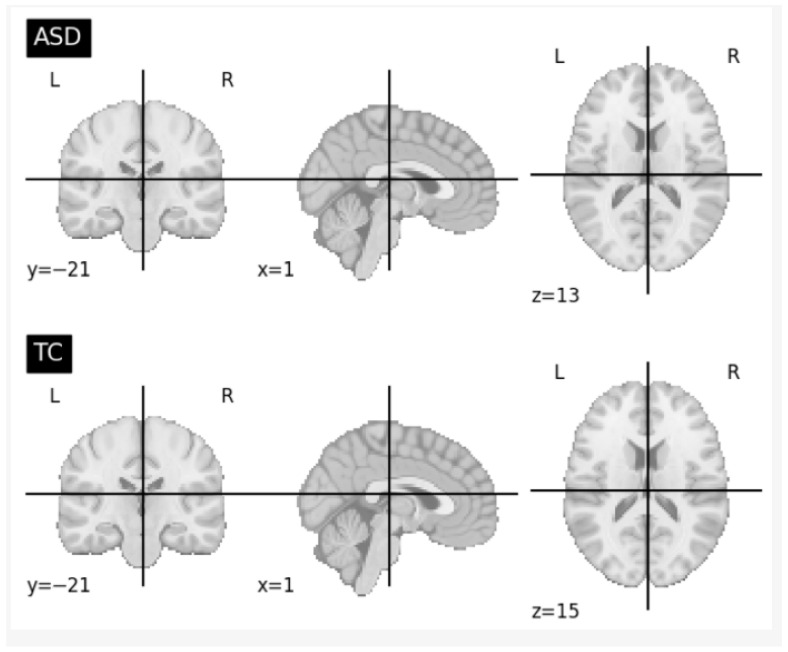
Visualization of Mean Functional Activation Across Orthogonal Planes for fMRI Preprocessing.

For spatial aggregation, slices within each time frame were averaged using the nilearn.image.mean_img function, resulting in a compact 3D representation for each time point. Importantly, this averaging operation was performed within individual time frames only and did not eliminate the temporal dimension of the fMRI data. The resulting time-ordered sequence of averaged 3D volumes was subsequently used for temporal modeling. Voxel intensities were then normalized to ensure uniformity and to reduce inter-subject variability arising from differences in scanning parameters. Figure 6 illustrates the resulting 3D fMRI representations across the axial, sagittal, and coronal planes.

**Figure 7:**
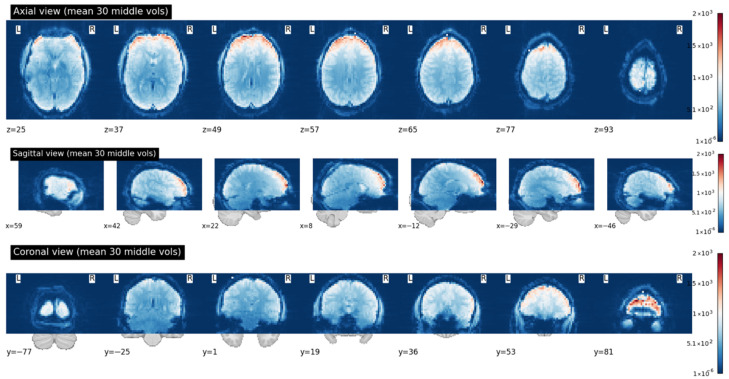
Visualization of Mean Functional Activity Focusing on the Central Brain Slices.

In addition, different slice retrieval strategies were explored to analyze their impact on model performance. Specifically, subsets of 10, 30, and 50 middle slices were extracted from the 4D fMRI data, followed by spatial averaging and normalization. Figure 7 presents representative images of ASD and typically developing control subjects derived from 30 middle slices across the three anatomical planes. Furthermore, an alternative configuration using all slices except the first and last 10 was examined to minimize non-informative or artifact-prone regions commonly observed at the beginning and end of fMRI scans. This step ensured retention of the most informative slices while reducing noise introduced during scan initialization and termination.

Following spatial aggregation and normalization, the fMRI data for each subject are represented as a time-ordered sequence of 3D brain volumes, with each volume corresponding to a single time point. This sequential representation preserves the temporal evolution of functional brain activity throughout the scan. To effectively model temporal dependencies and capture both past and future contextual information, a Bidirectional Long Short-Term Memory (Bi-LSTM) network is employed. The Bi-LSTM processes the sequence of averaged 3D volumes across time, enabling the learning of temporal patterns associated with ASD-related functional dynamics. This design ensures that spatial information is compactly encoded at each time point, while explicitly modeling temporal relationships across successive frames.

### 4.4. Baseline Algorithms

We selected five baselines, namely the Vision Transformer [31], Swin Transformer [32], Temporal Convolutional Network [33], 3D Convolutional Neural Network [34], and Dual-Branch Attention-Pruned Graph Neural Network [35]. The baselines were selected to provide a comprehensive comparison across both transformer-based and traditional deep learning frameworks for MRI-based investigations into ASD. The specifications and training protocols for each baseline are described below for transparency and reproducibility, as in Table 4. The first baseline, Vision Transformer, aimed to model long-range dependencies across MRI slices by treating each volume as a sequence of non-overlapping image patches. Each patch was 16 × 16 for two-dimensional slices and 16 × 16 × 16 for volumetric data. The model comprises twelve transformer encoder layers, each with 12 self-attention heads and an embedding dimension of 768. The MLP expansion ratio was set to 4, and layer normalization was applied before the attention and feed-forward stages. GELU is utilized as the activation function. The ADAM optimizer has a starting learning rate of 0.0001 and hyperparameters of β_1_ = 0.9 and β_2_ = 0.999. A warm-up schedule is used for the first 10 epochs, followed by a cosine annealing schedule. A batch size of 16 was used for the 2D input and 4 for the 3D input, with a dropout rate of 0.1. A weight decay of 0.01 was used as a regularization term, and the model was trained until the validation loss stopped decreasing for 500 consecutive epochs. The classification layer consisted of a softmax activation and cross-entropy loss for the binary classification of the TC and ASD classes.

The second baseline, the Swin Transformer, used a hierarchical representation with shifted-window attention to capture both local and global contextual cues from the MRI data. Input slices were split into 4-by-4 patches, with four hierarchical stages of depth 2, 2, 6, and 2. The base embedding dimension was 96, which was doubled after each down-sampling stage. Attention windows were 7 × 7 for the 2D input and 7 × 7 × 7 for the 3D input. The first hierarchical stage used three attention heads, which progressively increased in number in later stages. The activation function used GELU with an MLP ratio of 4. The ADAMW optimizer, with a weight decay of 0.05 and an initial learning rate of 0.00005, was used.

The third baseline, the Temporal Convolutional Network (TCN), was developed to capture sequential and temporal dependencies among MRI-derived features, such as dynamic connectivity and slice-wise temporal series. The model consisted of eight residual blocks, each comprising dilated causal convolutions with the dilation rate doubling in each subsequent block. Each convolution employed a kernel size of three and one hundred twenty-eight filters. Residual connections that utilized one-by-one convolutions were utilized to match the dimensions. The activation function was a ReLU, and a dropout rate of 0.2 was used within blocks to prevent overfitting. Weight normalization was also applied to stabilize the training process.

Furthermore, the ADAM optimizer was employed with a learning rate of 0.0003 and a weight decay of 0.0001. The learning rate was decreased by 0.1 if the validation loss plateaued after 10 epochs, and the batch size during training was set to 32. The model was trained for up to 100 epochs with early stopping, and the final prediction was made using a softmax classifier with cross-entropy loss.

The fourth baseline, a 3D Convolutional Neural Network (CNN), learned volumetric spatial dependencies across MRI voxels. The architecture consisted of six convolutional blocks, each utilizing a three-by-three-by-three kernel, followed by batch normalization and LeakyReLU activation with a negative slope of 0.01. The feature channels increased incrementally from 32 to 64, then to 128, and finally to 256. Global average pooling was applied before the last fully connected layer, and dropout with a rate of 0.4 was applied before classification. L2 regularization with a coefficient of 0.0005 was performed on all convolutional weights. Training was conducted with the ADAM optimizer (initial learning rate of 0.00001) and batch sizes of 8 for down-sampled data and 2 for full 3D volumes. The learning rate decayed by 0.1 during validation, but the improvement did not persist for 10 epochs. Training continued for up to 120 epochs with early stopping. Cross-entropy [36,37] was used as the classification loss, and mild affine transformations were applied for data augmentation.

The fifth baseline, Dual-Branch Attention-Pruned Graph Neural Network, leveraged brain connectivity information from MRI by forming graphs in which each node represented a region of interest and edges represented either functional or structural connectivity. The first branch used graph attention layers with 8 attention heads and a hidden dimension of 64 per head; the second branch used three spectral graph convolution layers with a hidden dimension of 256. The branches were combined using attention aggregators, with attention pruning by removing edges with refined attention weights below 0.05 to increase sparsity and focus on the most significant edges. ReLU activation was implemented after each graph layer with node-wise batch normalization. Subsequently, the fused representation was passed through two fully connected layers with a dropout rate of 0.3, then fed to the final softmax classifier. We used ADAM optimizer with a learning rate of 0.0002, a weight decay of 0.0005, and a batch size of sixteen. The learning rate decayed by 0.1 when the validation stagnated for ten epochs. This was performed for 200 epochs, utilizing class-weighted cross-entropy to mitigate class imbalance.

### 4.5. Performance Metrics

In assessing the performance of sMRI and fMRI in autism classification, evaluative studies evaluate both classification accuracy and the reliability of predicting a diagnosis. The evaluation metrics most often used in these types of studies are Accuracy, Precision, Recall/Sensitivity, Specificity, and F1 score [38]. Accuracy measures the fraction of subjects the algorithm correctly classified, while precision indicates the true proportion of ASD instances correctly predicted among all instances designated as ASD. Recall indicates the model’s ability to correctly predict every diagnostic case of ASD (without missing any cases), and specificity quantifies the true proportion of normal controls (NC) who were correctly identified. The F1 score is the harmonic mean of precision and recall, offering a balanced trade-off between precision and sensitivity. The mathematical representations of the applied metrics are given in Equations (35)–(38).

(35) $$Accuracy=\frac{TP+TN}{TP+FP+FN+TN}$$

(36) $$Precision=\frac{TP}{FP+TP}$$

(37) $$Recall=\frac{TP}{TP+FN}$$

(38) $$F-Measure=2\times \frac{Presision.Recall}{Presision+Recall}$$

In addition to these conventional measures, two progressive assessment elements: (1) Focal Loss, and (2) Receiver Operating Characteristic (ROC) curve were used to ensure a thorough assessment of the model’s diagnostic reliability. The Focal loss function was implemented to improve the model’s stability while also remaining sensitive to uncertain or borderline samples, even in a balanced dataset. The Focal Loss (FL) function [39] modifies the standard cross-entropy function as follows:

(39) $$FL\left(p_{t}\right)=-\alpha_{t}{\left(1-p_{t}\right)}^{\gamma }log(p_{t})$$

where $p_{t}$ is the predicted probability for the true class, and $\alpha_{t}$ = 0.25 balances the classes’ weights, while *γ* = 2 controls the emphasis on difficult-to-classify samples. The ROC curve [40] is a graphical method that illustrates how well a classification model performs across various decision thresholds. The ROC curve is plotted with the True Positive Rate (TPR), also known as sensitivity, on the *y*-axis and the False Positive Rate (FPR) on the *x*-axis. The curve conveys a balance between correctly classifying ASD subjects (true positives) and incorrectly classifying normal controls (false positives).

### 4.6. Experimental Setup

The experiments in this research were performed on Google Colab Pro+, a cloud computing environment that offers more advanced GPU and TPU resources, enabling the development and implementation of deep learning models more effectively. We selected the runtime with the NVIDIA A100 GPU to take advantage of the 83 GB of system memory, 40 GB of GPU RAM, 500 processing units, and 166 GB of disk space with rapid deep learning model training, optimization, and classification accuracy ranging from 30 min to 5 h based on the size of the data and the depth of the model. The Keras library’s implementation and model training are designed to be both flexible and easily deployable across other deep learning frameworks. Pre-processing of the MRI modalities was performed beforehand using the Nilearn, Nibabel, PIL, and NumPy libraries during this phase of the study on deep learning models. Nilearn and Nibabel libraries are used for visualizing, analyzing, and manipulating MRI modalities. Moreover, the Keras, NumPy, Nibabel, PIL, and Nilearn libraries were employed in a computational and analytical framework to handle and process MRI data for model training.

## 5. Experimental Results

The experimental evaluation involves four comparative scenarios to provide a comprehensive assessment of the proposed framework. (1) The proposed multi-view approach is compared to different methods operating on each view (axial, coronal, sagittal) with the sMRI data. (2) The same approach is performed with the fMRI data for a temporal–spatial analysis. (3) Various methods are compared utilizing the same data with multi-view fusion, to the hierarchical multi-view framework on the sMRI. (4) The same multi-view comparison is made with the fMRI data to assess robustness to temporal variability. These four cases collectively establish the superiority and generalization ability of the proposed model, as discussed in detail in the subsequent subsections.

### 5.1. ASD Classification Results from sMRI (Individual Brain View vs. Multiview)

From Table 5, the comparative evaluation across the axial, coronal, and sagittal planes shows distinct inter-model differences in focal loss, accuracy, and generalization patterns as a function of the number of slices. The ViT architecture exhibits moderate yet consistent improvement across all three planes as the number of slices increases, with accuracy in the axial plane increasing from 0.7034 with one slice to 0.7638 with fifty slices, while the focal loss decreases from 0.6427 to 0.5623. This same pattern was observed in both coronal (0.6942→0.7694) and sagittal (0.6898→0.7581) slices, suggesting that a deeper spatial representation helps learn the global context. However, all precision and recall scores (≈0.74 and ≈0.75 for higher slices) remained low, suggesting that ViT could learn global cortical representations well but struggled with local morphological anomalies—most likely due to patch-based embedding loss.

The Swin Transformer provides a better-balanced, spatially consistent presentation. In axial view, accuracy increases from 0.7241 to 0.7814 and results in a drop in the focal loss from 0.6295 to 0.5492. Coronal and sagittal accuracy results follow suit, showing similar performance with a total coronal accuracy of 0.7758 and 0.7724 in the sagittal view, respectively, in the fifty-slice representation. The approximate precision and recall of approximately 0.79 and 0.76 across the slice counts indicate that the Swin hierarchical attention mechanism leverages and maintains inter-slice continuity. The TCN has the advantage of learning sequential dependencies across slice stacks. The TCN axial accuracy steadily increases from 0.7294 to 0.7856, correlating with a decrease in focal loss from 0.6019 to 0.5287. The coronal and sagittal accuracy scores provide supportive evidence at 0.7828 and 0.7762 per plane, respectively, confirming the robustness of the model in a temporal perspective for feature extractability. Moreover, the improvement in recall rates is faster than that of precision (0.7423 to 0.7912 in the axial view), which may indicate that background noise issues surfaced as TCN focused on the very fine volumetric progression. Furthermore, the STN exhibited smooth F1-scores across all three planes at or near 0.78, indicating reliability for slice-sequential inter-feature propagation and a better generalized model that approached equal contextual dependency without inducing overfitting.

The 3D Convolutional Neural Network (3D-CNN) demonstrates a distinct volumetric advantage. Axial accuracy increases from 0.7425 at one slice to 0.8263 at fifty, along with a decrease in focal loss from 0.5708 to 0.4936. Coronal and sagittal views exhibit an identical pattern to the axial gains, concluding with final values of 0.8185 and 0.8168, respectively. The model’s F1-score peaks at 0.8109 in the axial view, indicating strong three-dimensional spatial integration in this representation. However, the gains for this model plateau at 50 slices, suggesting that the model loses representational novelty once sufficient volumetric context is established. Still, 3D-CNN demonstrates nearly uniform precision and recall in all planes, indicating a computationally expensive, but structurally consistent baseline.

The Dual-Branch Attention-Pruned Graph Neural Network (DB-APGNN) demonstrated the best overall performance compared to the baseline. The dual-branch attention mechanism strengthens relational reasoning of neighboring cortical regions and prunes graph connections to maintain efficiency. In the axial plane, focal loss drops from 0.5274 to 0.4578, while accuracy increases from 0.7769 to 0.8538, and the F1-score peaks at 0.8409. Like the axial findings, comparable benefits are seen in the coronal (0.7691→0.8482 accuracy) and sagittal (0.7634→0.8457 accuracy) planes.

The proposed Hierarchical Multi-View Deep Learning Framework significantly surpasses all comparative models on evaluation metrics. Focal loss decreased from 0.4876 at one slice to 0.3982 at fifty slices, while accuracy increased significantly from 0.8243 to 0.9019. The corresponding precision, recall, and F1 Scores of 0.9085, 0.8927, and 0.9005 were also the highest reported in the study’s results. The multi-view approach proposed differed from single-plane models by incorporating hierarchical fusion of [axial, coronal, and sagittal] representations that jointly modeled complementary spatial cues across planes. This gain across planes (ΔAccuracy ≈ +0.08 to +0.09 compared to DB-APGNN) shows that the enhanced gain is sensitive to structures that discriminate cortical-related irregularities associated with ASD-related irregularities in a multi-directional manner.

### 5.2. ASD Classification Results from fMRI (Individual Brain View vs. Multiview)

Table 6 reveals clear cross-model and cross-plane differences in performance for fMRI-based autism classification, attenuated by the temporal noise and spatial heterogeneity of fMRI signals. The Vision Transformer (ViT) displayed limited generalizability and moderate sensitivity to slice depth. In the axial plane, accuracy improved from 0.6541 at one slice to 0.7157 when using fifty slices, with the focal loss showing a corresponding decrease from 0.6948 to 0.6182. This 6.2% overall improvement in accuracy reflects increased contextualization of information between adjacent slices; however, precision (0.7039) and recall (0.6861) remain below 0.71, indicating erratic feature localization. Similar improvements were also seen in the coronal (0.6472→0.7064) and sagittal views (0.6398→0.7031) with the F1-score peaking at 0.6949. Upon reflecting on improvements in accuracy across models, ViT maintained a fairly high focal loss value for each of the imaging planes (≈0.63–0.71), consistently indicating the difficulty in capturing more nuanced dynamics of activations due to its attention formulation, which is primarily based on patch sizes. The Swin Transformer improves spatial coherence by leveraging hierarchical attention for localized representation learning. Classification accuracy increased from 0.6785 to 0.7426 in the axial view, while the coronal accuracy went from 0.6674 to 0.7342, and the focal loss decreased nearly 0.07 from 9 to 14 depth increments. Precision went from 0.6627 to 0.7289, while recall went from 0.6414 to 0.7103, leading to a gain of 10.3% in the F1 score (0.6518→0.7195). The maximum accuracy in the sagittal view was slightly lower (0.7291), and the maximum F1 score was also slightly lower (0.7044), suggesting that Swin’s spatial attention was not as effective with temporally elongate activations that are common in sagittal slices. Overall, Swin was observed to outperform ViT by about 2–3% for most metrics, reinforcing the idea that localized self-attention enhances learning by mitigating the impact of noise, as occurs in fMRI signals. The Temporal Convolutional Network (TCN) offers a distinct advantage in learning temporal patterns. When displayed across one slice, axial accuracy to hold a level of 0.6891, but when displayed across fifty slices, axial accuracy improved to 0.7516 with associated recall levels adjusting from 0.6956 to 0.7624 and the F1-score increasing from 0.6833 to 0.7516. The coronal and sagittal planes exhibited similar trends, with recorded accuracies of 0.7458 and 0.7407, respectively. A focal loss decrease of nearly 0.07 across all four orientations indicates that the model converged reasonably quickly, leveraging temporal dependency memory. The TCN sees an overall precision and recall range (≈0.74–0.75), providing evidence that learning models effectively extracted temporal features without overfitting. The stability of each plane F1-scores (0.7431–0.7516) provides a signal that the TCN consistently maintains the ability to extract dynamic activation sequences, which is particularly important for facilitating learning in fMRI.

The 3D Convolutional Neural Network (3D-CNN) has strong volumetric representation power. Accuracy improves from 0.7065 to 0.7931 in the axial plane, and focal loss decreases from 0.6172 to 0.5429. Both precision and recall achieve balanced performance of 0.7842 and 0.7741, respectively, resulting in an F1-score of 0.7791. The performances in the coronal and sagittal planes increase by about a similar amount, achieving accuracies of 0.7869 and 0.7812, respectively. These nearly uniform improvements across the axial, coronal, and sagittal planes (ΔAccuracy ≈ +0.08) exemplify the potential of 3D spatial kernels to effectively exploit temporal and spatial dependencies in fMRI. Focal loss is reduced by approximately 12% across all planes, indicating the model’s stability in convergence. However, this gain diminishes at higher slice counts, signaling that the efficiency of spatial encoding is reached once the volumetric features are sufficiently dense.

The Dual-Branch Attention-Pruned Graph Neural Network (DB-APGNN) outperforms the baseline across all three planes. Axial accuracy increased from 0.7398 to 0.8236 while focal loss dropped from 0.5786 to 0.5039. Precision and recall at 50 slices were 0.8165 and 0.8041, respectively, resulting in an F1 score of 0.8103. Both Coronal and Sagittal performance were slightly lower (accuracies 0.8162 and 0.8126), indicating spatial robustness to minimal orientation bias. Moreover, cross-plane focal loss variance (±0.02) was the lowest among all baselines, demonstrating the network’s ability to preserve connectivity features despite fMRI-induced temporal distortion.

The Proposed Hierarchical Multi-View Deep Learning Framework significantly outperforms all other approaches on all metrics. The focal loss shows a consistent drop from 0.5338 across one slice to 0.4437 across fifty slices, signifying a 16.9% reduction indicative of increased convergence stability. Accuracy improves from 0.7935 to 0.8893, which is 6.6% larger than that of DB-APGNN. The precision improves to 0.8978, with recall peaking at 0.8829, yielding the highest F1-score of 0.8903. The precision-recall values are nearly symmetrical, indicating balanced discrimination while maintaining low false positives or missed activations. The hierarchical multi-view fusion extends beyond the single-view networks to include spatial cues derived from the axial, coronal, and sagittal planes. The multi-plane 3D-CNN method shows an improvement of 0.07–0.09 in accuracy compared to the 3D-CNN using only one plane. The cross-plane integrates subtle, distributed patterns of activation associated with functional atypicality in ASD. The networks show the lowest focal loss and the highest F1 score, representing improved optimization stability and spatiotemporal adaptation.

**Figure 8:**
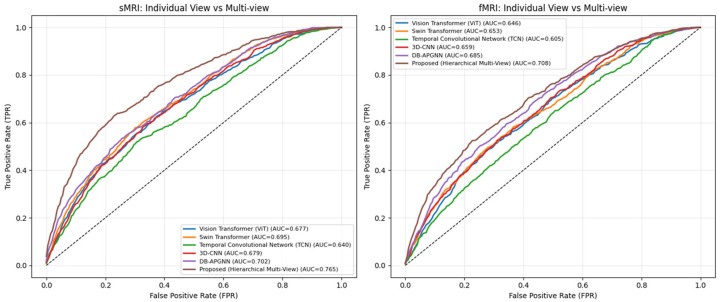
Comparative ROC Analysis of the Proposed Hierarchical Framework in Single-View vs. Multi-View Scenarios. Here dotted line shows random guess case performed by the classifier.

Figure 8 depicts two sets of ROC plots, with sMRI on the left and fMRI on the right, each with six methods. The resulting curves exhibit smooth, gradual transitions across the full false positive rate range, confirming that performance evaluation is not influenced by discretization or plotting artifacts. For sMRI, it attains an AUC of 0.765, representing an absolute improvement of approximately 6.3% over the strongest baseline (DB-APGNN, AUC 0.702), and larger margins over transformer-based and temporal models (0.640–0.695). For fMRI, the proposed method achieves an AUC of 0.708, outperforming DB-APGNN (0.685) and other baselines (0.605–0.659), resulting in approximately 2–3% improvement under more challenging functional imaging conditions. Importantly, the AUC values remain within a moderate range (≈0.60–0.77), indicating that the observed gains reflect improved feature separability rather than unrealistically perfect discrimination.

### 5.3. ASD Classification Results from sMRI (Multiview vs. Multiview)

The multi-view observations in Table 7 reveal a clear hierarchical improvement in performance across all architectures as the number of slices increases, as evidenced by the consistent reduction in focal loss and concurrent increases in accuracy, precision, recall, and F1 score. In the case of ViT, there was an accuracy improvement from 0.7425 at one slice to 0.8023 at fifty slices, with focal loss decreasing from 0.6034 to 0.5192. Precision and recall improved from 0.7316 and 0.7142 to 0.7981 and 0.7856, respectively, resulting in an F1 score of 0.7918.

The Swin Transformer achieves more consistent learning of spatial features, reducing focal loss from 0.5829 to 0.4968 and increasing accuracy from 0.7618 to 0.8264 and the F1 score from 0.7403 to 0.8055. Precision and recall ultimately reveal a narrow gap, from 0.018 to 0.016, indicating a more balanced classification. The Temporal Convolutional Network appears to benefit the most from temporal continuity across slices, with accuracy improving from 0.7746 to 0.8354, focal loss improving from 0.5587 to 0.4789, and with recall (0.7818 → 0.8368) consistently exceeding precision (0.7642 → 0.8235), all leading to the most consistent of recall trends amongst all other non-graph baselines and producing F1 = 0.8301. The 3D Convolutional Neural Network (3D-CNN) effectively leverages volumetric consistency, achieving 0.8617 in accuracy, 0.8476 in F1-score, and a 13.7% decrease in focal loss (0.5236 to 0.4518). The precision and recall are very tightly aligned (0.8516, compared to 0.8437 for recall), which helps confirm minimal bias and generalization. The DB-APGNN enables the model to leverage relational reasoning further, improving accuracy from 0.8164 to 0.8807 and reducing focal loss from 0.4895 to 0.4189. For precision (0.8748), recall (0.8627), and thus the F1-score (0.8687), the proposed method improved by 6.8% compared to the 3D-CNN baseline. Overall, the multi-view variants of all baseline methods demonstrated noticeable improvements over their respective single-view performances; however, their global results remained inferior to those achieved by the proposed framework.

### 5.4. ASD Classification Results from fMRI (Multiview vs. Multiview)

The results of the different views presented in Table 8 indicate that each model improves consistently with increasing slice depth, further suggesting that incorporating more spatial and temporal information into the parameter estimates may aid in classifying stable outputs. The Vision Transformer (ViT) achieved moderate improvements; accuracy improved from 0.6981 to 0.7583, while focal loss improved from 0.6418 to 0.5672, and both precision and recall improved to 0.7496 and 0.7349, respectively, which, in turn, improved the F1 score to 0.7421, but while stable, the patch-based design keeps mean spatial consistency lower than other models. The Swin Transformer model demonstrated greater stability, achieving an accuracy of 0.7768 and an F1 score of 0.7537 on slices with a depth of 50. This is reflected in a 12% improvement in focal loss stability, as evidenced by the precision–recall balance, which improves from 0.7642 to 0.7436. The TCN model effectively capitalized on temporal dependencies, improving accuracy from 0.7262 to 0.7849 and recall from 0.7358 to 0.7896, while achieving a reliable F1 score of 0.7818.

The 3D-CNN model successfully captured volumetric cues, achieving an accuracy range of 0.7386 to 0.8124 and a lower focal loss of 0.4932, while maintaining precision and recall reasonably aligned at 0.8023 versus 0.7905, confirming effective spatial generalization. The DB-APGNN model outperformed all other baselines, achieving an accuracy of 0.8413 and an F1 score of 0.8287, with a relatively strong precision–recall balance of 0.8341 versus 0.8234. This is accompanied by a 13% improvement in focal loss, suggesting its strong analytical ability to learn relational problem sets across regions. The proposed hierarchical multi-view model achieved the best overall performance, with specified accuracies ranging from 0.8017 to 0.8847, precision ranging from 0.8086 to 0.8923, and recall ranging from 0.7949 to 0.8769. Its final F1-score of 0.8845 and lowest focal loss (0.4267) indicate superior optimization and balanced detection capability. Similarly, to the sMRI modality, the multi-view variants of all baseline methods also exhibited substantial performance gains over their individual brain views. However, their overall results still lagged behind the superior performance achieved by the proposed framework.

**Figure 9:**
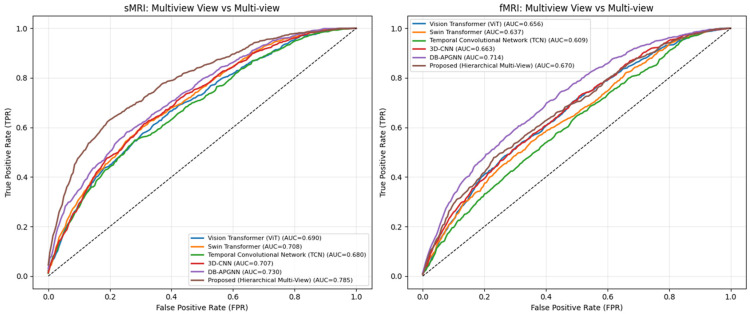
Comprehensive Comparative ROC Analysis of Multi-View Frameworks for sMRI and fMRI Classification. Here dotted line shows random guess case performed by the classifier.

Figure 9 illustrates the comparative performance of baseline methods versus the proposed framework in a multi-view vs. multiview scenario across sMRI and fMRI modalities. For sMRI, the proposed hierarchical multi-view framework achieves the highest discriminative performance with an AUC of 0.785, outperforming DB-APGNN (0.730), Swin Transformer (0.708), 3D-CNN (0.707), Vision Transformer (0.690), and TCN (0.680). It corresponds to an absolute improvement of approximately 5–10% over competing multi-view baselines, indicating that hierarchical fusion of complementary views enhances class separability beyond simple aggregation strategies.

In the fMRI setting, overall performance is lower, reflecting the higher variability and temporal complexity of functional signals. Nevertheless, the proposed method achieves an AUC of 0.670, remaining competitive with DB-APGNN (0.714) and outperforming transformer-based and temporal baselines (0.609–0.656). While DB-APGNN exhibits a higher AUC in this modality, the proposed framework demonstrates more balanced performance across both structural and functional views, suggesting improved robustness when handling heterogeneous multi-view information.

Importantly, the AUC values for both modalities remain within a moderate range (≈0.60–0.79), indicating realistic class overlap rather than near-perfect discrimination. These results show that hierarchical multi-view modeling consistently improves sensitivity across operating points and that its benefits are particularly pronounced for sMRI, where complementary spatial views provide stronger and more stable discriminative cues.

### 5.5. Ablation Studies

To clarify the contribution of individual components in the sMRI and fMRI branches, statistical comparisons revealing consistent performance trends are summarized in Table 9 and Table 10. For the sMRI branch, the full hierarchical multi-view configuration using 50 slices achieves an average accuracy of 90.19 ± 0.12% with the lowest focal loss (0.3982), significantly outperforming alternative configurations. Statistical significance is assessed using a paired Wilcoxon signed-rank test applied to subject-wise cross-validation results, yielding *p* < 0.001, which reflects consistent performance gains across folds rather than an inflated sample size. When the number of slices is reduced, accuracy and F1-score decrease by approximately 1.3% and 2.6% for the 25-slice and 10-slice models, respectively, while focal loss increases to 0.4163 and 0.4415.

The one-slice model exhibits a pronounced performance drop of nearly 8% relative to the baseline (*p* < 0.001). Single-view models (axial, sagittal, and coronal) achieve lower accuracies of 84.1–85.1%. To assess the effect of view diversity across these configurations, a one-way ANOVA reveals a significant impact on overall accuracy (F = 19.42, *p* < 0.01). Removing hierarchical feature fusion or Grad-CAM guidance results in F1-score reductions of 3.9% and 3.12%, respectively (*p* < 0.05), indicating their contributions to multi-scale representation and spatial interpretability. The reduced-depth model achieves an accuracy of 88.12 ± 0.18, which does not differ significantly from the baseline (*p* > 0.05), suggesting that moderate compression preserves much of the discriminative capacity.

For the fMRI branch, the full configuration attains an accuracy of 88.93 ± 0.15 with the lowest focal loss (0.4437). Replacing the LSTM with temporal averaging reduces accuracy to 83.42 ± 0.25 (*p* < 0.001) and increases focal loss to 0.5018, indicating the importance of temporal modeling. Substituting the LSTM with a 1-D convolution results in a smaller accuracy reduction of approximately 2.2% (*p* < 0.05). Disabling attention pooling decreases the F1-score by 4.4%, while removing ROI mapping or motion correction produces the largest reductions (6.3% and 7.9%, respectively; *p* < 0.001). These effects remain statistically significant after Bonferroni correction, highlighting the role of spatial summarization and preprocessing in temporal reliability. The cross-interaction layer improves recall by 2.7% relative to simple feature concatenation (*p* < 0.05), supporting its contribution to multimodal integration.

### 5.6. Cross-Dataset Generalization

The cross-site evaluation on the UCLA dataset reveals a markedly non-uniform performance distribution, reflecting the realistic impact of scanner variability and limited sample size. As shown in Table 11, the full model using 50 slices attains an accuracy of 68.40% ± 0.92, but exhibits a pronounced imbalance between precision (74.10%) and recall (61.85%). This disparity suggests that, under cross-site conditions, the model becomes more conservative in its predictions, favoring specificity over sensitivity.

Interestingly, the 25-slice configuration shows contrasting behavior, with recall (69.80%) exceeding precision (62.35%), suggesting a greater tendency to detect ASD cases at the cost of increased false positives. This asymmetric precision–recall trade-off highlights the sensitivity of cross-site performance to slice selection and emphasizes that accuracy alone is insufficient to characterize model behavior. For the 10-slice configuration, performance degradation is accompanied by increased variability and a sharp decline in recall (55.20%), despite relatively high precision (71.90%). It suggests unstable feature representation when the spatial context is insufficient, leading to inconsistent decision boundaries. The single-slice setting exhibits the weakest and most unstable performance, with accuracy dropping to 58.30% ± 1.88 and the highest focal loss (0.8026), reflecting poor confidence calibration and increased optimization difficulty.

### 5.7. Research Limitations

Although the proposed multi-view hierarchical framework shows good accuracy and robustness in both sMRI and fMRI modalities, there remain limitations to be overcome.

1. The research uses data from a limited number of imaging sites, which may limit generalizability across heterogeneous first-time experiences in both populations and acquisition protocols.
2. The analysis framework assumes spatial and temporal (or non-spatial-scanning) consistency across modalities, which may not be maintained in clinical datasets.
3. The fixed slice counts and the ROI mappings may not fully capture each subject’s anatomical and functional variations.
4. The computational complexity and memory requirements of the model may compromise scalability when applied to large cohorts or used in real-time clinical settings.
5. External validation on independent datasets was not conducted, thereby limiting the study’s statistical generalizability.

### 5.8. Computational Complexity and Efficiency Evaluation

**Figure 10:**
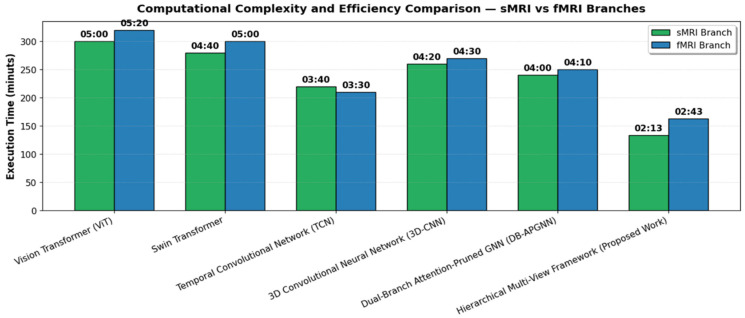
Comparative Analysis of Computational Efficiency and Inference Time for Dual-Modal Branches.

The proposed framework is intentionally designed as a lightweight alternative to full 3D volumetric processing. Rather than exhaustively computing over entire MRI volumes, the model operates on a representative subset of slices extracted from large 3D data. This design choice significantly reduces computational overhead while preserving diagnostically relevant information, thereby enabling efficient training and inference. The reported runtime comparisons are therefore intended to highlight the practical efficiency of the proposed slice-based strategy, rather than to suggest a strictly equalized input-size benchmark across all baseline models. Figure 10 compares computational complexity and efficiency, revealing a clear hierarchy in performance across all evaluated models for both the sMRI and fMRI branches. It is clear that the Vision Transformer had the slowest inference time, taking 5 min for sMRI and 5 min 20 s for fMRI, reflecting the heavy computational burden of self-attention operation common in transformer-based models, and the Swin Transformer slightly improved on those times, taking 4 min 40 s for sMRI and 5 min for fMRI, because of its windowed attention model, which does reduce the burden of computation modestly; TCN took the times down even further with run times at 3 min and 40 s for sMRI and 3 min and 30 s for fMRI, suggesting it was more efficient in the local processing of temporal data. The 3D CNN had run times of 4 min and 20 s for sMRI and 4 min and 30 s for fMRI, which implies a steady cost of depth for spatial representation; however, it was still more efficient than the transformer-based models because it maintained a more structured approach. The DB-APGNN’s efficiency was attributed to its attention sparsity and pruning, as it took 4 min for sMRI and 4 min and 10 s for fMRI.

Interestingly, the proposed Hierarchical Multi-View Deep Learning Framework outperformed all baselines, with execution times of just 2 min 13 s for sMRI and 2 min 43 s for fMRI, making it the most computationally efficient in this study. This improvement means that execution time was reduced by nearly 50 percent compared with the transformer baseline networks and improved by around 35 percent compared with the GNN baseline networks. The small difference in execution times between fMRI and sMRI was attributed to the use of an additional temporal encoding layer in the fMRI methods’ branch.

## 6. Conclusions and Future Scope

The proposed hierarchical multi-view deep learning framework demonstrated consistent advantages across both sMRI and fMRI modalities. Specifically, for the sMRI branch, the complete configuration achieved an accuracy of 90.19 ± 0.12%, precision of 90.85 ± 0.16%, recall of 89.27 ± 0.19%, and an F1 score of 90.05 ± 0.14%, with the lowest focal loss of 0.3982. Likewise, the fMRI branch achieved an accuracy of 88.93 ± 0.15%, precision of 89.78 ± 0.18%, recall of 88.29 ± 0.20%, and an F1 score of 89.03 ± 0.17%, with a focal loss of 0.4437. It demonstrates that the proposed model can effectively utilize spatial and temporal information while providing systematically higher performance than transformer, convolutional, and graph-based architectures across modalities, both in terms of accuracy and computational complexity. The model’s multi-view optimization and hierarchical encoding enable strong generalization while maintaining low inference time and, ideally, minimal focal loss, demonstrating its robustness for multimodal neuroimaging approaches. In the future, this framework can be extended to accommodate multi-site and multi-protocol datasets, thereby increasing cross-population robustness. Improvements to the framework can include adaptive temporal synchronization and dynamic attention alignment, as well as models of causal interpretability, enabling the transparent learning of neural representations. The framework can be applied to longitudinal studies or to studies of specific diseases (e.g., autism, Alzheimer’s, and Parkinson’s) to support claims of diagnostic utility. Furthermore, GPU–TPU optimized deployment offers opportunities for real-time clinical integration, thereby making this framework an ideal foundation for scalable, efficient, and interpretable neuroimaging analytics.

## Figures and Tables

**Table 1:** Summary of the latest research studies in ASD diagnosis.

Reference	Problem Statement	Proposed Approach	Used Modality	Research Gaps
[20]	Small fMRI datasets and poor generalizability of ASD detection	Deep neural network using functional connectivity features from multiple atlases (CC200, AAL, BASC, Power)	fMRI	Reliance on a specific atlas (e.g., BASC) may impact generalizability across datasets.
[21]	Lack of reliable biomarkers for early ASD diagnosis	Deep learning framework using fMRI and sMRI with a custom block for dataset expansion	fMRI + sMRI	Performance is insufficient for clinical applicability
[22]	Difficulty in accurate early diagnosis of ASD using MRI	DM-ResNet framework combining FCM-GMM segmentation, VGG-16 feature extraction, and Dwarf Mongoose optimized ResNet	MRI	High accuracy (99.83%) raises concerns about robustness and generalizability across diverse clinical datasets
[23]	Limited interpretability and reliability in ASD diagnosis from brain connectivity features	Two-step deep learning model with residual attention network for spatiotemporal features and GCN for connectivity-based classification	fMRI	Reliance on specific connectivity hypotheses (STS-visual cortex) may limit broader applicability and generalization
[24]	High spatio-temporal complexity and limited generalization ability	Functional connectivity features and F-score-based feature selection	fMRI	Variability in study design and preprocessing may affect reproducibility and clinical use.
[25]	Robust ASD classification using ABIDE II dataset	Cortex extraction→parcellation→correlation matrix→recursive feature elimination→SVM	fMRI (ABIDE II)	Reliance on parcellation schemes and radiomic features limits adaptability to different cohorts
[26]	Early detection of ASD from high-dimensional resting-state fMRI	CNN architecture with convolution, normalization, dropout, dense layers	Resting-state fMRI	Homogeneous sample population, limiting generalizability
[27]	Glymphatic function and white matter integrity in ASD children	Multi-parametric MRI indices (aDTI-ALPS, FA, CSF volume, PVS volume) + AutoGluon model	Multi-parametric MRI	Retrospective design and small validation cohort across two centers

**Table 2:** Overview of the NYU dataset derived from the ABIDE I repository.

Dataset	Subjects	ASD	Control	Imaging Modalities	Comments
NYU	184	79	105	rs-fMRI,T1-weighted MRI	Balanced dataset, widely used baseline in ABIDE I studies

**Table 3:** The number of slices (i.e., 2D images) generated from sMRI using multi-slice generation.

Site	3D sMRI	Brain View	Slices/Plane	Train Set (80%)		Test Set (20%)	
				ASD	NC	ASD	NC
NYU	ASD 79, TC 105	Axial	1	64	84	15	21
NYU	ASD 79, TC 105	Coronal	1	64	84	15	21
NYU	ASD 79, TC 105	Sagittal	1	64	84	15	21
NYU	ASD 79, TC 105	Axial	10	640	840	150	210
NYU	ASD 79, TC 105	Coronal	10	640	840	150	210
NYU	ASD 79, TC 105	Sagittal	10	640	840	150	210
NYU	ASD 79, TC 105	Axial	50	3200	4200	750	1050
NYU	ASD 79, TC 105	Coronal	50	3200	4200	750	1050
NYU	ASD 79, TC 105	Sagittal	50	3200	4200	750	1050

**Table 4:** Detailed configuration of baseline algorithms.

Algorithm	Architectural Summary	Training Configuration	Regularization & Loss
Vision Transformer (ViT)	12-layer encoder with 12 self-attention heads and embedding dimension of 768; input divided into 16 × 16 (2D) or 16 × 16 × 16 (3D) patches	ADAM optimizer (β_1_ = 0.9, β_2_ = 0.999), learning rate 0.0001, batch size 16 (2D)/4 (3D), warm-up for 10 epochs followed by cosine decay	Dropout rate 0.1, weight decay 0.01 (L2 regularization), cross-entropy loss
Swin Transformer	Hierarchical structure with four stages [2,6]; shifted window attention (7 × 7 for 2D/7 × 7 × 7 for 3D); base embedding 96	ADAMW optimizer, learning rate 0.00005, batch size 8 (2D)/2 (3D), 5-epoch warm-up and cosine decay schedule	Dropout 0.3, weight decay 0.05, cross-entropy loss
Temporal Convolutional Network (TCN)	8 dilated residual blocks with kernel size 3 and channel width 128; causal convolutions for temporal modeling	ADAM optimizer, learning rate 0.0003, batch size 32, decay factor 0.1 after 10 stagnant epochs	Dropout 0.2, L2 penalty 0.0001, cross-entropy loss
3D Convolutional Neural Network (3D-CNN)	6 convolutional blocks (3 × 3 × 3 kernel) with channel progression 32→256; global average pooling before classification	ADAM optimizer, learning rate 0.00001, batch size 8 (down-sampled)/2 (full 3D), 10-epoch decay schedule	Dropout 0.4, L2 = 0.0005, cross-entropy loss
Dual-Branch Attention-Pruned Graph Neural Network (DB-APGNN)	Two-branch design with GAT layers (8 heads, 64-dim) and 3 spectral GCN layers (256-dim); attention pruning below 0.05	ADAM optimizer, learning rate 0.0002, batch size 16, 10-epoch adaptive decay	Dropout 0.3, L2 = 0.0005, weighted cross-entropy loss

**Table 5:** Comparative performance of baseline and proposed models across individual brain views of baseline methods vs. the multiview of the proposed approach with sMRI modality.

Method	Brain Plane	No. Slices	Focal Loss	Accuracy	Precision	Recall	F1-Score
Vision Transformer (ViT)	Axial	1 slice	0.6427	0.7034	0.6889	0.6712	0.6795
		10 slices	0.6018	0.7475	0.7421	0.7328	0.7374
		50 slices	0.5623	0.7638	0.7651	0.7484	0.7566
	Coronal	1 slice	0.6539	0.6942	0.6816	0.6664	0.6732
		10 slices	0.6156	0.7317	0.7394	0.7142	0.7265
		50 slices	0.5678	0.7694	0.7615	0.7472	0.7542
	Sagittal	1 slice	0.6583	0.6898	0.6751	0.6637	0.6693
		10 slices	0.6114	0.7352	0.7284	0.7196	0.7239
		50 slices	0.5697	0.7581	0.7638	0.7415	0.7525
Swin Transformer	Axial	1 slice	0.6295	0.7241	0.7317	0.7074	0.7193
		10 slices	0.5836	0.7543	0.7685	0.7356	0.7516
		50 slices	0.5492	0.7814	0.7936	0.7589	0.7758
	Coronal	1 slice	0.6384	0.7115	0.7163	0.6932	0.7045
		10 slices	0.5937	0.7486	0.7551	0.7274	0.7409
		50 slices	0.5519	0.7758	0.7824	0.7542	0.7680
	Sagittal	1 slice	0.6412	0.7029	0.7138	0.6875	0.7004
		10 slices	0.5981	0.7443	0.7521	0.7238	0.7376
		50 slices	0.5567	0.7724	0.7809	0.7513	0.7658
Temporal Convolutional Network (TCN)	Axial	1 slice	0.6019	0.7294	0.7175	0.7423	0.7297
		10 slices	0.5698	0.7625	0.7486	0.7693	0.7588
		50 slices	0.5287	0.7856	0.7769	0.7912	0.7839
	Coronal	1 slice	0.6183	0.7172	0.7078	0.7349	0.7211
		10 slices	0.5741	0.7581	0.7442	0.7653	0.7547
		50 slices	0.5376	0.7828	0.7754	0.7876	0.7814
	Sagittal	1 slice	0.6135	0.7209	0.7132	0.7311	0.7220
		10 slices	0.5772	0.7498	0.7417	0.7578	0.7497
		50 slices	0.5403	0.7762	0.7693	0.7826	0.7758
3D Convolutional Neural Network (3D-CNN)	Axial	1 slice	0.5708	0.7425	0.7321	0.7242	0.7281
		10 slices	0.5293	0.7927	0.7845	0.7756	0.7800
		50 slices	0.4936	0.8263	0.8172	0.8047	0.8109
	Coronal	1 slice	0.5834	0.7332	0.7281	0.7169	0.7224
		10 slices	0.5437	0.7851	0.7734	0.7683	0.7708
		50 slices	0.5059	0.8185	0.8103	0.7976	0.8039
	Sagittal	1 slice	0.5861	0.7267	0.7194	0.7073	0.7132
		10 slices	0.5468	0.7815	0.7719	0.7624	0.7671
		50 slices	0.5093	0.8168	0.8076	0.7932	0.8003
Dual-Branch Attention-Pruned GNN (DB-APGNN)	Axial	1 slice	0.5274	0.7769	0.7846	0.7614	0.7728
		10 slices	0.4892	0.8273	0.8221	0.8094	0.8157
		50 slices	0.4578	0.8538	0.8462	0.8357	0.8409
	Coronal	1 slice	0.5396	0.7691	0.7764	0.7561	0.7661
		10 slices	0.4975	0.8214	0.8136	0.8049	0.8092
		50 slices	0.4639	0.8482	0.8394	0.8297	0.8345
	Sagittal	1 slice	0.5418	0.7634	0.7712	0.7506	0.7607
		10 slices	0.5013	0.8189	0.8093	0.7995	0.8043
		50 slices	0.4675	0.8457	0.8369	0.8256	0.8312
Hierarchical Multi-View Deep Learning Framework (Proposed Work)	Multi-view (Axial, Coronal, Sagittal)	1 slice	0.4876	0.8243	0.8324	0.8196	0.8260
		10 slices	0.4415	0.8748	0.8816	0.8674	0.8744
		50 slices	0.3982	0.9019	0.9085	0.8927	0.9005

**Table 6:** Comparative performance of baseline and proposed models across individual brain views of baseline methods vs. the multiview of the proposed approach with fMRI modality.

Method	Brain Plane	No. Slice	Focal Loss	Accuracy	Precision	Recall	F1-Score
Vision Transformer (ViT)	Axial	1 slice	0.6948	0.6541	0.6438	0.6189	0.6311
		10 slices	0.6624	0.6885	0.6723	0.6594	0.6658
		50 slices	0.6182	0.7157	0.7039	0.6861	0.6949
	Coronal	1 slice	0.7025	0.6472	0.6321	0.6103	0.6210
		10 slices	0.6743	0.6746	0.6618	0.6459	0.6538
		50 slices	0.6328	0.7064	0.6935	0.6762	0.6847
	Sagittal	1 slice	0.7104	0.6398	0.6252	0.6023	0.6135
		10 slices	0.6817	0.6679	0.6534	0.6396	0.6464
		50 slices	0.6386	0.7031	0.6875	0.6698	0.6786
Swin Transformer	Axial	1 slice	0.6732	0.6785	0.6627	0.6414	0.6518
		10 slices	0.6336	0.7149	0.7018	0.6745	0.6879
		50 slices	0.5994	0.7426	0.7289	0.7103	0.7195
	Coronal	1 slice	0.6898	0.6674	0.6521	0.6318	0.6417
		10 slices	0.6527	0.7028	0.6894	0.6632	0.6761
		50 slices	0.6135	0.7342	0.7217	0.7015	0.7115
	Sagittal	1 slice	0.6951	0.6618	0.6482	0.6246	0.6362
		10 slices	0.6634	0.6987	0.6853	0.6589	0.6718
		50 slices	0.6267	0.7291	0.7154	0.6936	0.7044
Temporal Convolutional Network (TCN)	Axial	1 slice	0.6529	0.6891	0.6714	0.6956	0.6833
		10 slices	0.6198	0.7237	0.7118	0.7273	0.7194
		50 slices	0.5842	0.7516	0.7411	0.7624	0.7516
	Coronal	1 slice	0.6631	0.6758	0.6641	0.6783	0.6711
		10 slices	0.6294	0.7112	0.7026	0.7194	0.7109
		50 slices	0.5956	0.7458	0.7334	0.7531	0.7431
	Sagittal	1 slice	0.6715	0.6682	0.6548	0.6716	0.6631
		10 slices	0.6373	0.7061	0.6927	0.7135	0.7030
		50 slices	0.6028	0.7407	0.7282	0.7479	0.7379
3D Convolutional Neural Network (3D-CNN)	Axial	1 slice	0.6172	0.7065	0.6951	0.6897	0.6924
		10 slices	0.5763	0.7594	0.7517	0.7398	0.7457
		50 slices	0.5429	0.7931	0.7842	0.7741	0.7791
	Coronal	1 slice	0.6247	0.6993	0.6868	0.6805	0.6836
		10 slices	0.5864	0.7532	0.7459	0.7326	0.7392
		50 slices	0.5512	0.7869	0.7775	0.7671	0.7723
	Sagittal	1 slice	0.6315	0.6917	0.6792	0.6723	0.6757
		10 slices	0.5931	0.7491	0.7416	0.7273	0.7343
		50 slices	0.5573	0.7812	0.7729	0.7604	0.7666
Dual-Branch Attention-Pruned GNN (DB-APGNN)	Axial	1 slice	0.5786	0.7398	0.7513	0.7228	0.7368
		10 slices	0.5397	0.7921	0.7867	0.7692	0.7778
		50 slices	0.5039	0.8236	0.8165	0.8041	0.8103
	Coronal	1 slice	0.5892	0.7312	0.7408	0.7171	0.7288
		10 slices	0.5486	0.7837	0.7782	0.7635	0.7708
		50 slices	0.5125	0.8162	0.8083	0.7958	0.8020
	Sagittal	1 slice	0.5941	0.7254	0.7342	0.7118	0.7229
		10 slices	0.5538	0.7784	0.7726	0.7574	0.7649
		50 slices	0.5179	0.8126	0.8042	0.7909	0.7974
Hierarchical Multi-View Deep Learning Framework (Proposed Work)	Multi-view (Axial, Coronal, Sagittal)	1 slice	0.5338	0.7935	0.8019	0.7894	0.7956
		10 slices	0.4861	0.8524	0.8617	0.8483	0.8549
		50 slices	0.4437	0.8893	0.8978	0.8829	0.8903

**Table 7:** Comparative performance of baseline and proposed models across multiview slices of baseline methods vs. the proposed approach with sMRI modality.

Method	No. Slices	Focal Loss	Accuracy	Precision	Recall	F1-Score
Vision Transformer (ViT)	1 slice	0.6034	0.7425	0.7316	0.7142	0.7228
	10 slices	0.5618	0.7786	0.7742	0.7597	0.7669
	50 slices	0.5192	0.8023	0.7981	0.7856	0.7918
Swin Transformer	1 slice	0.5829	0.7618	0.7493	0.7315	0.7403
	10 slices	0.5374	0.7987	0.7864	0.7681	0.7771
	50 slices	0.4968	0.8264	0.8138	0.7974	0.8055
Temporal Convolutional Network (TCN)	1 slice	0.5587	0.7746	0.7642	0.7818	0.7729
	10 slices	0.5173	0.8092	0.7971	0.8119	0.8044
	50 slices	0.4789	0.8354	0.8235	0.8368	0.8301
3D Convolutional Neural Network (3D-CNN)	1 slice	0.5236	0.7884	0.7816	0.7725	0.7770
	10 slices	0.4871	0.8325	0.8239	0.8174	0.8206
	50 slices	0.4518	0.8617	0.8516	0.8437	0.8476
Dual-Branch Attention-Pruned GNN (DB-APGNN)	1 slice	0.4895	0.8164	0.8241	0.8027	0.8132
	10 slices	0.4528	0.8546	0.8493	0.8351	0.8421
	50 slices	0.4189	0.8807	0.8748	0.8627	0.8687
Hierarchical Multi-View Deep Learning Framework (Proposed Work)	1 slice	0.4876	0.8243	0.8324	0.8196	0.8260
	10 slices	0.4415	0.8748	0.8816	0.8674	0.8744
	50 slices	0.3982	0.9019	0.9085	0.8927	0.9005

**Table 8:** Comparative performance of baseline and proposed models across multiview slices of baseline methods vs. the proposed approach with fMRI modality.

Method	No. Slices	Focal Loss	Accuracy	Precision	Recall	F1-Score
Vision Transformer (ViT)	1 slice	0.6418	0.6981	0.6853	0.6627	0.6738
	10 slices	0.6035	0.7324	0.7247	0.7085	0.7165
	50 slices	0.5672	0.7583	0.7496	0.7349	0.7421
Swin Transformer	1 slice	0.6249	0.7143	0.7032	0.6818	0.6923
	10 slices	0.5827	0.7491	0.7398	0.7169	0.7282
	50 slices	0.5476	0.7768	0.7642	0.7436	0.7537
Temporal Convolutional Network (TCN)	1 slice	0.5985	0.7262	0.7137	0.7358	0.7246
	10 slices	0.5613	0.7586	0.7485	0.7619	0.7551
	50 slices	0.5251	0.7849	0.7742	0.7896	0.7818
3D Convolutional Neural Network (3D-CNN)	1 slice	0.5634	0.7386	0.7294	0.7201	0.7247
	10 slices	0.5285	0.7819	0.7712	0.7628	0.7670
	50 slices	0.4932	0.8124	0.8023	0.7905	0.7963
Dual-Branch Attention-Pruned GNN (DB-APGNN)	1 slice	0.5258	0.7697	0.7768	0.7521	0.7643
	10 slices	0.4891	0.8108	0.8043	0.7902	0.7972
	50 slices	0.4556	0.8413	0.8341	0.8234	0.8287
Hierarchical Multi-View Deep Learning Framework (Proposed Work)	1 slice	0.5338	0.7935	0.8019	0.7894	0.7956
	10 slices	0.4861	0.8524	0.8617	0.8483	0.8549
	50 slices	0.4437	0.8893	0.8978	0.8829	0.8903

**Table 9:** Statistical ablation results of the sMRI branch showing the effect of volumetric depth, multi-view fusion, and hierarchical encoding on classification performance.

No.	Configuration	Accuracy (%)	Precision (%)	Recall (%)	F1-Score (%)	Focal Loss
1	Full Hierarchical Multi-View (50 slices)	90.19 ± 0.12	90.85 ± 0.16	89.27 ± 0.19	90.05 ± 0.14	0.3982
2	Multi-View (25 slices)	88.91 ± 0.15	89.43 ± 0.17	88.25 ± 0.18	88.84 ± 0.16	0.4163
3	Multi-View (10 slices)	87.48 ± 0.21	88.16 ± 0.18	86.74 ± 0.24	87.44 ± 0.20	0.4415
4	Multi-View (1 slice)	82.43 ± 0.26	83.24 ± 0.22	81.96 ± 0.28	82.60 ± 0.24	0.4876
5	Axial Only	84.11 ± 0.25	84.78 ± 0.23	83.65 ± 0.29	84.16 ± 0.25	0.4748
6	Sagittal Only	84.93 ± 0.23	85.34 ± 0.24	84.12 ± 0.28	84.73 ± 0.24	0.4720
7	Coronal Only	85.08 ± 0.24	85.71 ± 0.22	84.81 ± 0.27	85.25 ± 0.25	0.4695
8	Feature Hierarchy Removed	86.08 ± 0.19	86.91 ± 0.20	85.42 ± 0.23	86.16 ± 0.21	0.4534
9	Grad-CAM Guidance Disabled	87.01 ± 0.22	87.54 ± 0.24	86.67 ± 0.25	87.10 ± 0.23	0.4462
10	Data Augmentation Removed	85.76 ± 0.27	86.11 ± 0.28	85.42 ± 0.30	85.76 ± 0.28	0.4589
11	Reduced Convolutional Depth	88.12 ± 0.18	88.47 ± 0.20	87.95 ± 0.22	88.21 ± 0.19	0.4312

**Table 10:** Statistical ablation results of the fMRI branch highlighting the impact of temporal modeling, attention pooling, and ROI mapping on overall accuracy.

No.	Configuration	Accuracy (%)	Precision (%)	Recall (%)	F1-Score (%)	Focal Loss
1	Full model (50 slices)	88.93 ± 0.15	89.78 ± 0.18	88.29 ± 0.20	89.03 ± 0.17	0.4437
2	Multi-View (25 slices)	87.42 ± 0.17	88.03 ± 0.20	86.95 ± 0.19	87.49 ± 0.18	0.4622
3	Multi-View (10 slices)	85.24 ± 0.22	86.17 ± 0.23	84.83 ± 0.26	85.49 ± 0.23	0.4861
4	Multi-View (1 slice)	79.35 ± 0.27	80.19 ± 0.25	78.94 ± 0.29	79.56 ± 0.26	0.5338
5	LSTM Replaced with 1D Convolution	86.91 ± 0.20	87.14 ± 0.21	86.28 ± 0.23	86.70 ± 0.21	0.4629
6	LSTM Replaced with Temporal Averaging	83.42 ± 0.25	83.75 ± 0.24	82.91 ± 0.28	83.33 ± 0.26	0.5018
7	Attention Pooling Replaced by Global Average Pooling	84.62 ± 0.23	85.01 ± 0.22	84.17 ± 0.25	84.59 ± 0.23	0.4887
8	Attention Replaced by Self-Weight Averaging	86.14 ± 0.21	86.67 ± 0.22	85.91 ± 0.24	86.28 ± 0.22	0.4713
9	ROI Mapping Removed	82.77 ± 0.24	83.10 ± 0.25	82.34 ± 0.26	82.72 ± 0.25	0.5094
10	ROI Atlas-Based (Fixed Regions)	85.93 ± 0.20	86.32 ± 0.21	85.10 ± 0.23	85.70 ± 0.21	0.4758
11	Motion Correction Removed	81.18 ± 0.29	81.37 ± 0.27	80.52 ± 0.30	80.94 ± 0.28	0.5235
12	Cross Interaction Layer Removed	86.23 ± 0.19	86.71 ± 0.20	85.94 ± 0.22	86.31 ± 0.20	0.4683
13	Temporal Down sampling (Half Resolution)	84.48 ± 0.24	85.02 ± 0.25	83.90 ± 0.26	84.45 ± 0.24	0.4939
14	Added Gaussian Noise (Signal Distortion Test)	80.84 ± 0.31	81.23 ± 0.30	80.17 ± 0.33	80.67 ± 0.31	0.5292

**Table 11:** Cross-site classification performance on the UCLA dataset (*n* = 16) using different slice-based configurations with the model trained on the NYU site and evaluated without fine-tuning.

No.	Configuration	Accuracy (%)	Precision (%)	Recall (%)	F1-Score (%)	Focal Loss
1	Full model (50 slices)	68.40 ± 0.92	74.10 ± 1.05	61.85 ± 1.18	67.41 ± 0.98	0.6487
2	Multi-View (25 slices)	66.10 ± 1.10	62.35 ± 1.28	69.80 ± 1.34	65.86 ± 1.19	0.6732
3	Multi-View (10 slices)	63.75 ± 1.46	71.90 ± 1.62	55.20 ± 1.71	62.46 ± 1.54	0.7218
4	Multi-View (1 slice)	58.30 ± 1.88	59.85 ± 1.95	56.10 ± 2.04	57.92 ± 1.90	0.8026

## Data Availability

The data presented in this study are available in the Autism Brain Imaging Data Exchange (ABIDE) repository at http://fcon_1000.projects.nitrc.org/indi/abide/, accessed on 23 February 2026.

## Abbreviations

The following abbreviations are used in this manuscript:

CNN	Convolution Neural Network
ASD	Autism Spectrum Disorder
MRI	Magnetic Resonance Imaging
sMRI	Structural Magnetic Resonance Imaging
fMRI	Functional Magnetic Resonance
LSTM	Long Short-Term Memory
ABIDE	Autism Brain imaging Data Exchange
NYU	New York University
ML	Machine Learning
DL	Deep Learning
SVM	Support Vector Machine
Grad-CAM	Gradient- weighted Class Activation Mapping
DM- ResNet	Dwarf Mongoose -Residual Network
FCM	Fuzzy C-Means
GMM	Gaussian Mixture Model
VGG- 16	Visual Geometry Group
AUC	Area Under the Curve
GCN	Graph Convolutional Network
ROI	Region of Interest
3D-HCNN	3-Dimensional Hierarchical Convolutional Neural Network
ReLU	Rectified Linear Unit
MLP	Multilayer Perceptron
LN	Layer Normalization
GELU	Gaussian Error Linear Unit
3D-CNNs	3D Convolutional Neural Networks
NIfTI	Neuroimaging Informatics Technology Initiative
TCN	Temporal Convolutional Network
ADAM	Adaptive Moment Estimation
ViT	Vision Transformer
DB-APGNN	Dual-Branch Attention-Pruned Graph Neural Network
ROC	Receiver Operating Characteristic
TPR	True Positive Rate
FPR	False Positive Rate
GPU	Graphics Processing Unit
TPU	Tensor Processing Unit
